# Determinants of Cell-to-Cell Variability in Protein Kinase Signaling

**DOI:** 10.1371/journal.pcbi.1003357

**Published:** 2013-12-05

**Authors:** Matthias Jeschke, Stephan Baumgärtner, Stefan Legewie

**Affiliations:** 1 Institute of Molecular Biology (IMB), Mainz, Germany; North Carolina State University, United States of America

## Abstract

Cells reliably sense environmental changes despite internal and external fluctuations, but the mechanisms underlying robustness remain unclear. We analyzed how fluctuations in signaling protein concentrations give rise to cell-to-cell variability in protein kinase signaling using analytical theory and numerical simulations. We characterized the dose-response behavior of signaling cascades by calculating the stimulus level at which a pathway responds (‘pathway sensitivity’) and the maximal activation level upon strong stimulation. Minimal kinase cascades with gradual dose-response behavior show strong variability, because the pathway sensitivity and the maximal activation level cannot be simultaneously invariant. Negative feedback regulation resolves this trade-off and coordinately reduces fluctuations in the pathway sensitivity and maximal activation. Feedbacks acting at different levels in the cascade control different aspects of the dose-response curve, thereby synergistically reducing the variability. We also investigated more complex, ultrasensitive signaling cascades capable of switch-like decision making, and found that these can be inherently robust to protein concentration fluctuations. We describe how the cell-to-cell variability of ultrasensitive signaling systems can be actively regulated, e.g., by altering the expression of phosphatase(s) or by feedback/feedforward loops. Our calculations reveal that slow transcriptional negative feedback loops allow for variability suppression while maintaining switch-like decision making. Taken together, we describe design principles of signaling cascades that promote robustness. Our results may explain why certain signaling cascades like the yeast pheromone pathway show switch-like decision making with little cell-to-cell variability.

## Author Summary

Cells sense their surroundings and respond to soluble factors in the extracellular space. Extracellular factors frequently induce heterogeneous responses, thereby restricting the biological outcome to a fraction of the cell population. However, the question arises how such cell-to-cell variability can be controlled, because some cellular systems show a very homogenous response at a defined level of an extracellular stimulus. We derived an analytical framework to systematically characterize the cell-to-cell variability of intracellular signaling pathways which transduce external signals. We analyzed how heterogeneity arises from fluctuations in the total concentrations of signaling proteins because this is the main source of variability in eukaryotic systems. We find that signaling pathways can be highly variable or inherently invariant, depending on the kinetic parameters and the structural features of the cascade. Our results indicate that the cell-to-cell variability can be reduced by negative feedback in the cascade or by signaling crosstalk between parallel pathways. We precisely define the role of negative feedback loops in variability suppression, and show that different aspects of the dose-response curve can be controlled, depending on the feedback kinetics and site of action in the cascade. This work constitutes a first step towards a systematic understanding of cell-to-cell variability in signal transduction.

## Introduction

**Figure 1:**
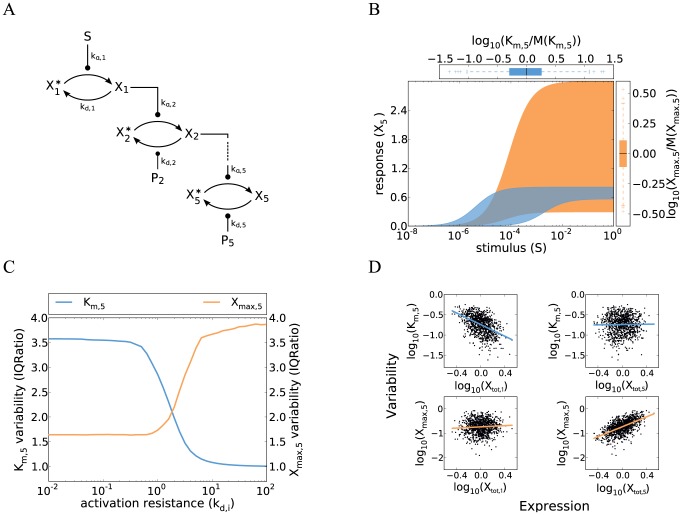
Cell-to-cell variability in a minimal model of a gradual kinase cascade. **A** Schematic representation of a five-step kinase cascade (S…extracellular stimulus; 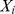 and 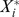
…active and inactive kinases, respectively; 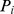
…phosphatases; 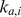 and 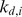
…phosphorylation and dephosphorylation rate constants, respectively). **B** Cell-to-cell variability simulations confirm strong heterogeneity in the gradual kinase cascade. Nine signaling protein concentrations (5 kinases, 4 phosphatases) were sampled from log-normal distributions (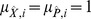; coefficient of variation = 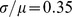), and the dose-response curve was simulated using Eqs. 3 and 4 for a set of 1000 sampled protein concentrations. Low phosphatase activities were chosen to model a low activation resistance: 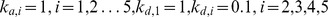 (Supplemental Table S1). The blue and orange areas are enclosed by the dose-response curves which yielded the minimal/maximal 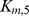 and 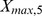, respectively. Box plots at the top and right side represent the distributions of 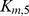 and 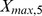, respectively (normalized by the population medians). These box plots indicate the median (middle of box), the first and third quartile (box edges), the data points that lie within a distance of 1.5 interquartile ranges from the lower and higher quartiles (whiskers) and extreme outliers (crosses). **C** The variabilities of 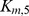 and 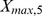 respond inversely to changes in kinetic parameter values. Cell-to-cell variability simulations (similar to panel **B**) were repeated for various activation resistances in the cascade which were tuned by simultaneously changing the phosphatase rate constants 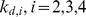 (x-axis). The variabilities of 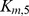 and 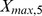 were analyzed for each parameter configuration (y-axis) and expressed as inter-quartile ratios (IQRatio = 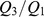 = ratio of the third quartile and the first quartile; related to the width of the box plots shown in **B**). High inter-quartile ratios imply high cell-to-cell variability while an IQRatio of 1 corresponds to no variability. Similar results are obtained when using the coefficient of variation as a measure of variability (Figure S1). **D** Upstream signaling protein fluctuations determine the pathway sensitivity (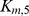) while downstream fluctuations control the maximal pathway activation (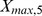). 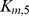 and 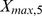 were calculated for each simulation run in panel **B** and related to the concentrations of the first and the terminal kinase in the same simulation. Each dot represents a simulation of a single cell, and the solid lines are linear fits to all points.

External stimuli typically induce cellular responses by binding to cell surface receptors. Intracellular signaling networks transduce the signal, ultimately triggering gene expression responses in the nucleus. The basic building blocks of eukaryotic signaling networks are protein kinase cascades (Figure 1A): The signaling proteins in the cascade act as enzymes (“kinases”) that catalyze the activation of downstream kinases by phosphorylation. Information is thus transmitted along the cascade by consecutive phosphorylation reactions (Figure 1A). The proto-typical example for such a signaling cascade is the conserved mitogen-activated protein kinase (MAPK) pathway which consists of three kinases (Raf, Mek, Erk) [1].

Signaling cascades can transduce information in different ways [2], [3]. The activity of the terminal kinase may quantitatively reflect the concentration of the extracellular stimulus, and the cascade is termed to behave gradually (or analog) in this case. Alternatively, the cascade may act as an ultrasensitive switch that responds in a digital (“all-or-none”) manner: low background signals are strongly dampened and rejected, while amplification and cellular commitment occur once a threshold stimulus is reached. Ultrasensitive signaling cascades therefore act as cellular decision making devices. Theoretical studies revealed that minimal models of multi-step protein kinase cascades show gradual dose-response behavior at steady state [4]. Ultrasensitive decision making requires additional regulation mechanisms which increase the steepness of the dose-response curve, e.g., strong enzyme saturation in the (de)phosphorylation reactions (“zero-order ultrasensitivity”), multisite phosphorylation, competitive inhibition, or positive feedback [3], [5].

The dose-response curve of a signaling pathway relates the signaling activity to the amount of extracellular stimulus applied. The dose-response curve of signaling pathways is typically sigmoidal in shape and can be quantitatively described by the so-called Hill equation (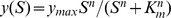, with 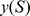 as the response to the stimulus 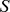). The half-maximal stimulus (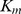) characterizes the stimulus concentration where the signal reaches 50% of its maximal activation level, and is thus a measure of the pathway sensitivity towards extracellular stimulation. The maximal activation level (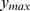) describes how strongly the terminal kinase can be activated upon very strong stimulation, thereby reflecting amplification or dampening potential of the cascade. The Hill coefficient 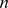 determines how steeply the pathway responds to external stimulation: the signaling cascade shows gradual behavior for 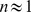, while ultrasensitive decision making is observed for 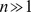. In the limit of very high 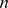 the dose-response approaches a step-function and the pathway acts as a digital switch with the threshold stimulus 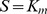.

Signaling networks show non-genetic variability, implying that the signaling activity can differ strongly between cells of a clonal population [6], [7]. Biological mechanisms underlying signaling variability include cell density effects [8] and cell-to-cell variability in signaling protein expression [6], [7], [9]. In the latter case, the stochasticity of protein biosynthesis indirectly hampers the precision of intracellular information transmission. An alternative source of variability may be the stochastic dynamics of signaling pathways operating at low molecule numbers [10]. Stochastic signaling fluctuations are typically fast compared to subsequent gene expression responses, and therefore should not impinge significantly on cellular decision-making. The variability of most signaling systems can therefore be understood by considering them as deterministic system with fluctuating initial signaling protein concentrations [6], [7], [9]. Single-cell measurements reveal that the level of each signaling protein differs by a factor of three among the cells of a clonal population [11], [12]. Thus, multi-component signaling systems may show strong variability, suggesting that regulation mechanisms exist which allow for variability suppression.

Cell-to-cell variability in the intracellular signaling pathway activity may be beneficial or deleterious depending on the biological system. Certain cellular responses such as apoptosis or differentiation should only be triggered in a subset of the cell population to maintain tissue homeostasis and to establish different cell lineages, respectively. The apoptosis and differentiation thresholds should thus be very different between individual cells and the system should exhibit strong variability [6], [13]. In cancer therapy, such strong heterogeneity may adversely affect the population responsiveness to drugs, thereby leading to incomplete killing of tumor cells [14]–[16]. Invariance of signaling thresholds is expected to be important in embryonic development: according to the so-called “French-flag model”, patterning is established by a single morphogen gradient that specifies multiple cell fates, each cell type requiring a different threshold morphogen concentration [17]. For sharp spatial boundaries to be established, signaling pathways that read of morphogen gradients should exhibit robust and invariant thresholds at which they respond. Similarly, a cell-to-cell invariant signaling threshold has been reported for yeast cells that sense positioning in an extracellular pheromone gradient [18], [19]. Low variability is also required for gradual signaling pathways which transduce information quantitatively. Taken together, the question arises how cellular systems are able to tune the variability of protein kinase signaling to ensure an appropriate response of the cell population.

In this work, we systematically characterize the cell-to-cell variability of protein kinase cascades. We focus on the dose-response behavior of signaling to investigate how synchronously a cell population responds to a change in a hormonal stimulus. We discuss how the variability can be actively modulated by parameter tuning, gene expression noise regulation or additional signaling mechanisms such as feedforward and feedback loops.

## Results

### 1 Rationale

This work focuses on the cell-to-cell variability of protein kinase cascades. We study the general features of eukaryotic signaling pathways, but also try to specifically answer the question why the yeast pheromone pathway shows switch-like decision making with little cell-to-cell variability [18], [19]. The pheromone pathway initiates the mating of two haploid yeast cells by triggering various cellular responses, one of which is the so-called shmoo, a cellular projection in the direction of the mating partner that primes for cell fusion [18], [20]. Dose-response experiments with exogenously added pheromones revealed that shmooing occurs at a similar pheromone concentration for all cells in the population, implying that the signaling pathway shows little cell-to-cell variability [18], [19], [21]: The transition from no shmooing to complete shmooing of the whole cell population occurred within a 2-fold range of pheromone concentrations in one study [18], while others reported that the required pheromone increase is 4-fold [19] or 5-fold [21]. In this paper, we analyze the dose-response curves of signaling pathways to understand how a coordinated response of the whole cell population at a particular stimulus concentration can be realized. We study simplified models of signaling cascades with five levels to reflect the main steps of pheromone signaling, i.e., pheromone binding to a transmembrane receptor, receptor-mediated G protein activation and signal transduction through a three-tiered MAPK cascade [22].

We characterize the dose-response behavior at steady state. Steady state simulations imply that we focus on sustained signaling upon long-term stimulation and neglect the temporal features of the signal such as duration or area-under-curve. Steady state simulations likely provide physiologically relevant insights, because many cell fate decisions require ongoing signaling pathway activity over several hours [23]. Fast signaling events such as phosphorylation and dephosphorylation typically occur on a time-scale of minutes, and are thus expected to reach a (quasi-)steady state shortly after external stimulation.

Signaling dose-response curves may increase gradually and reflect the concentration of the extracellular stimulus, or the signaling pathway may act as an ultrasensitive switch that responds in a digital (“all-or-none”) manner (see Introduction). The shmooing of yeast cells is an all-or-none response [18]. Contradictory evidence exist in whether or not digital decision making already occurs at the level of MAPK signaling [18], [21], [24], but the pathway likely exhibits a certain degree of ultrasensitivity [20]. In this paper, we employ a bottom-up approach and initially study minimal signaling models with gradual dose-response curves, before turning to more complex systems capable of ultrasensitive signal transduction.

Cell-to-cell variability is introduced into the models by assuming fluctuations in initial signaling protein expression levels. In contrast to previous studies on variability [25], we neglect the intrinsic stochasticity of signaling cascades (cf. Introduction), and analyze deterministic models of kinase signaling using the framework of ordinary differential equations (ODEs). Experimental studies suggest that all signaling protein concentrations vary simultaneously due to noise in protein biosynthesis rates [6], [7], [9]. Extrinsic noise sources, in particular signaling protein concentration fluctuations, are thought to be the main source of non-genetic variability in yeast pheromone signaling [26] and in mammalian signaling pathways [6], [7], [27]–[29]. We applied two complementary strategies to understand how signaling protein expression noise gives rise to signaling heterogeneity. First, explicit cell-to-cell variability simulations were performed. All signaling protein concentrations were sampled from uncorrelated log-normal distributions, and the ODE system was solved for each set of sampled concentrations, yielding distributions in signaling pathway activity. Secondly, one-dimensional sensitivity analyses revealed the impact of individual signaling protein concentrations: The ODE system was solved for varying levels of each signaling protein (keeping all other components constant). Signaling protein concentrations that had a strong impact on signaling pathway activity could be identified as major determinants of cell-to-cell variability.

Our results show that generic five-step protein kinase cascades exhibit much stronger cell-to-cell variability than the yeast pheromone pathway, unless certain robustness requirements are fulfilled.

### 2 Cell-to-cell variability of gradual protein kinase cascades

#### 2.1 Gradual protein kinase cascades show strong cell-to-cell variability

Multi-step signaling cascades show a gradual dose-response behavior if the response of each individual cascade level is gradual as well [3,30]. A minimal model of a gradual signaling cascade can be implemented by assuming that enzyme saturation in the phosphorylation and dephosphorylation reactions at each level are negligible [4].

The following ordinary differential equation describes the temporal evolution of the active kinase 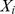:
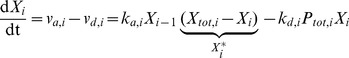
(1)


Each phosphorylation step is described as a reaction between the phosphorylated form 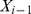 of kinase 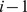 and the non-phosphorylated form 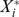 of a downstream kinase 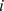 (Figure 1A). The corresponding phosphorylation rate is given by the term 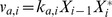 where 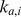 is the second-order rate rate constant for phosphorylation of the 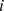-th kinase. Similarly, the dephosphorylation of the active form 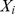 is described by the rate 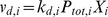, with the dephosphorylation rate constant and total phosphatase concentration designated as 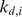 and 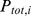, respectively [4]. Equation 1 takes into account that the total kinase concentration at each level is constant, i.e., 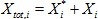.

The steady state activity of each cascade level describing the activity upon long-term stimulation can be calculated by assuming that the kinase concentrations do not change over time (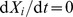).
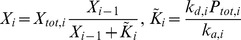
(2)


This expression relates the activity of the 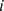-th kinase to that of its upstream activator 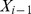, and therefore characterizes the local dose-response behavior of the cascade. It has the form of a Michaelis-Menten equation: Each cascade level may saturate if the kinase pool is fully phosphorylated (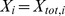) and half-maximal activation occurs when the kinase and phosphatase activities are equal 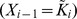.

The cellular response to stimulation is determined by the global dose-response curve which relates the activity of the terminal cascade level to the concentration of the extracellular stimulus *S*. By iteratively applying Eq. 2 and setting the stimulus to 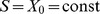, one derives for the global dose-response curve of a five-step cascade (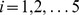)
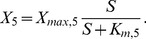
(3)


This Michaelis-Menten-like equation increases gradually for increasing concentrations of the stimulus 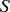, confirming that the minimal cascade model shows gradual dose-response behavior. The parameter 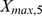 describes the maximal activation level of the pathway upon strong stimulation. 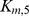 equals the stimulus concentration leading to half-maximal signaling, and thus reflects the pathway sensitivity to stimulation. 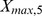 and 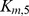 are lumped parameters that can be defined as
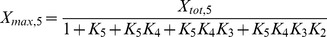
(4)

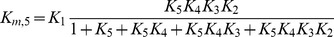




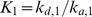
 is the dissociation constant of receptor-ligand binding, and the remaining 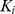 are proportional to the kinase and phosphatase concentrations in the cascade,
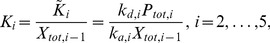
(5)To understand the cell-to-cell variability, we need to know how 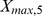 and 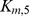 depend on the total kinase and phosphatase concentrations. We initially analyze cell-to-cell variability for the case of weak stimulation (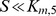) where the pathway dose-response curve in Eq. 3 can be approximated by the following linear equation:
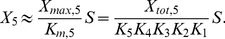
(6)


The signaling activity upon weak stimulation is thus determined by the product of five kinase concentrations (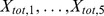) divided by the product of four phosphatase concentrations (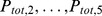). This implies that a weakly stimulated cascade exhibits strong cell-to-cell variability, because the product of fluctuating species shows much greater variability than either species alone. The total variance in the signaling output upon weak stimulation (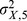) equals the sum over all signaling protein concentration variances (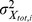 for kinases and 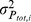 for phosphatases) (Supplemental Text S1).
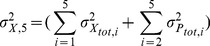
(7)


Two conclusions can be drawn from this equation concerning the regulation of variability: (i) the variability cannot be reduced significantly by lowering the expression noise of certain signaling proteins; instead, a simultaneous noise reduction of all species would be required: The cell-to-cell variability can be quantified using the inter-quartile ratio (IQ ratio) which expresses the difference of cells with high and low signaling activities by dividing the third and first quartiles of the distribution (see Methods). Assuming realistic protein concentration fluctuations in Eq. 6, the IQ ratio only drops from 4.1 to 3.8 if the noise of 1 out of 9 signaling protein concentrations is eliminated, implying that the cell-to-cell variability remains essentially unchanged. (ii) the variability does not depend on the choice of the kinetic parameter values 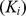. Thus, the weakly stimulated gradual signaling pathway always shows strong variability. Consistent with the expectation, we find that lesser variability may be observed upon strong stimulation 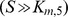 where 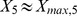 (see below).

The variability principles derived from the analytical model (Eq. 1–7) were confirmed by explicit cell-to-cell variability simulations. To this end, each of the nine protein concentrations in the cascade was sampled from a log-normal distribution with a coefficient of variation that matches the experimentally observed variability of eukaryotic protein expression [12]. Dose-response simulations were performed for each set of sampled protein concentrations, yielding cohorts of dose-response curves representing the cell population. Such a simulation is shown in Figure 1B, and cells with the highest and lowest 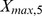 or 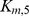 are highlighted by the shaded areas. These cell-to-cell variability simulations confirm that the minimal gradual protein kinase cascade generally shows pronounced variability, especially at low-level stimulation.

#### 2.2 A trade-off in controlling the variability of maximal pathway activation and pathway sensitivity

We investigated how the variabilities of the maximal pathway activation (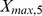) and the pathway sensitivity (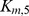) depend on the kinetic parameters in the cascade. 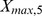 and 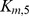 are fully described by the lumped parameters 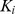 (Eq. 4). Each 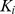 equals the phosphatase activity at a cascade level divided by the maximally possible kinase activity (Eq. 5). Thus, 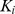 quantifies the tendency of a cascade level to be fully activated upon strong stimulation and can be considered as an activation resistance. A strong stimulus fully activates the pathway kinases only if all resistances are low (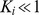).

We tuned the activation resistances (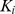) by simultaneously changing all phosphatase activities, and performed cell-to-cell variability simulations (Figure 1C). For low phosphatase activity at each level (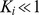), we observe little variance in the maximal pathway activation, because only the concentration of the terminal kinase matters (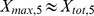). At the same time, the pathway sensitivity is determined by the product of multiple protein concentrations (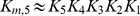), and therefore differs strongly between individual cells. In the opposite regime of high phosphatase activity at each level (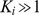), we find that the pathway sensitivity is completely invariant between cells. This is because the receptor level saturates before the subsequent cascade steps, implying that the dose-response curve of the terminal kinase is aligned to the half-maximal saturation point of receptor-ligand binding (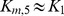). In this regime, the maximal activation level is, however, determined by all protein concentrations in the cascade and thus highly variable (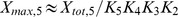). These simulations reveal that the variabilities of 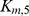 and 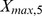 are inversely related. The drop in the variability of 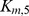 precisely matches the parameter range where the variability of 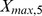 increases (Figure 1C). We show more generally in Supplemental Text S1 that 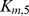 and 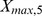 are inversely related for any parameter change in the signaling cascade. Thus, a trade-off exists in a simple gradual protein kinase cascade: either the pathway sensitivity or the maximal pathway activation can be made invariant by changing the kinetic parameter values. However, it is not possible to make 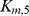 and 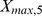 invariant at the same time.

The signaling variability could be reduced by lowering the expression noise of individual signaling proteins. We therefore investigated whether fluctuations in certain signaling protein concentrations have particularly strong impact on the variabilities of the maximal pathway activation (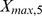) and the pathway sensitivity (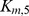). To this end, 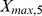 and 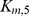 were related to the signaling protein expression levels in single cells (Figure 1D). Cells with a high expression level of the terminal kinase (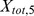) tend to have a higher maximal pathway output 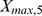 than cells harboring low levels of the terminal kinase. No such correlation is observed for the kinase concentration at the first cascade level (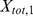). Thus, the downstream species tend to exert a stronger control on the maximal pathway output than the upstream species (see also Supplemental Text S1). In contrast, the pathway sensitivity is primarily determined by upstream species in the cascade: Cells tend to respond at lower ligand concentrations the higher the expression level of the first kinase (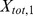) is, and the concentrations of the downstream species play a lesser role in this respect (Figure 1D, top row; Supplemental Text S1). Taken together, we find that the maximal output 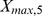 and the pathway sensitivity 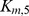 are controlled in a very different way. We show in Supplemental Text S1 that signaling protein concentrations with strongly control over 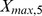 generally have lesser impact on 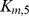 (and vice versa). Thus, while either 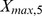 or 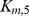 can be made invariant by reducing the expression noise of certain signaling proteins, it is not possible to achieve invariance for both features at the same time.

The trade-off in the regulation of 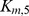 and 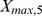 has important ramifications for the control of intracellular signaling: Intracellular signaling regulators or pharmacological inhibitors acting upstream in the cascade primarily regulate the pathway sensitivity, whereas downstream regulators predominantly affect the maximal pathway activation. The strong and parameter-independent dose-response variability suggests that the simple gradual model is unable reflect the invariant dose-response behavior of the yeast pheromone pathway (see Rationale): all cells of the population respond upon strong stimulation if the activation resistances are low (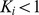), but then the pathway sensitivity fluctuations are high, and the most sensitive cells respond at a ∼100-fold lower stimulus concentration than the least sensitive cells (Figure 1B).

#### 2.3 Negative feedback regulation allows for the simultaneous invariance of maximal pathway activation and pathway sensitivity

Negative feedback regulation reduces the variability of biological systems [31–33]. In the following, we show that negative feedback resolves the above-mentioned robustness trade-off by simultaneously promoting the invariance of maximal pathway activation (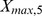) or pathway sensitivity (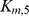).

**Figure 2:**
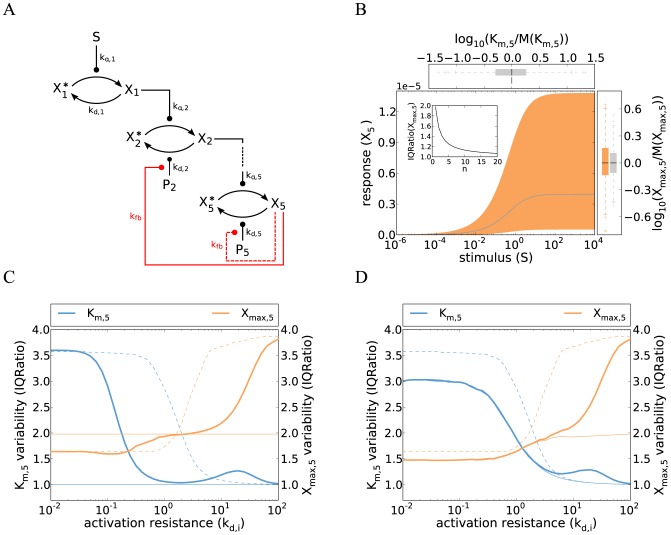
Cell-to-cell variability of kinase cascades with negative feedback. **A** Schematic representation of the five-step cascade with negative feedback acting upstream (red, solid) or downstream (red, dashed). 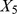 either activates the phosphatase of the second or the fifth level. **B** Cell-to-cell variability simulations confirm that negative feedback eliminates the variability of the pathway sensitivity (concepts similar to Figure 1B). Strong feedback was assumed and simulations were performed using Eq. 9 (parameters same as in Figure 1B; Supplemental Table S1). Colored box plots represent the 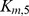 and 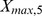 distribution of the feedback model, while gray box plots show the behavior of the reference feedback-less cascade (cf. Figure 1B). The inset shows that increasing the feedback cooperativity parameter 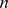 (Eq. 8) decreases 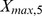 variability, measured as IQRatio (cf. Figure 1C). **C**–**D** Negative feedback abrogates the trade-off in 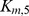 and 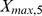 invariance. Cell-to-cell variability simulations (similar to panel **B**) were repeated for various parameter configurations for models with upstream feedback (**C**) or downstream feedback (**D**): activation resistances in the cascade were tuned by simultaneously changing the phosphatase rate constants 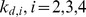 (x-axis). The variabilities of 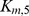 and 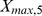 were analyzed using the IQRatio as in Figure 1C, and similar results are obtained using the coefficient of variation (Figure S2). 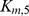 was defined as the stimulus for a half-maximal pathway activation. The behavior of a feedback model with limited feedback strength (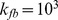 ; thick, solid lines) is compared to a feedback-less model (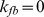; thin, dashed lines) and to a model with very strong feedback 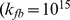; thin, solid lines). Simulations for moderate feedback strength (thick lines) were performed by numerically integrating the ODE systems (Eqs. 8 and 12), while the strong feedback calculations (thin solid lines) were done using analytical approximations (Eqs. 9 and 13).

In the yeast pheromone pathway, the terminal kinase promotes the deactivation of the pathway by negatively regulating the G protein level [22,34], and this negative feedback loop has been reported to control the pathway sensitivity to stimulation [34]. We implemented negative feedback in a gradual five-step protein kinase cascade by assuming that the final kinase (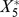) enhances the activity of the phosphatase at the second level (Figure 2A, solid red line). Most of the differential equations remain unchanged when compared to the basic model (Eq. 1), but the ODE for the second pathway level now reads:
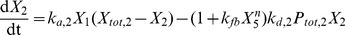
(8)


The rate of 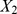 deactivation is multiplied by the term 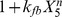 to reflect that the dephosphorylation is enhanced by 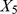. The parameter 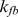 determines how strongly 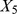 promotes the deactivation of 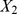. The exponent 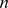 indicates a possible cooperativity of negative feedback regulation (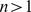: feedback with positive cooperativity). For pronounced feedback regulation, i.e., 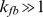, the steady state dose-response curve can be approximated by (Supplemental Text S1):
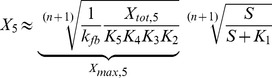
(9)


In line with previous studies, we find that the kinase cascade with negative feedback regulation exhibits a shallower dose-response curve than the feedback-less system, because the stimulus 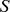 enters as the 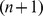-th root only [32,33,35]. The non-cooperative feedback system requires a ∼420-fold increase in the stimulus level to switch from 10% to 90% of the maximal activation level, while an 81-fold increase is sufficient in the corresponding feedback-less cascade (Eq. 3). Negative feedback therefore extends the gradual mode of quantitative information transmission to a large stimulus concentration range, and the effect is more pronounced for cooperative feedback regulation 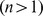.

Strong negative feedback reduces the cell-to-cell variability of the dose-response curve: The half-maximal stimulus of the cascade is proportional to the half-saturation point of receptor-ligand binding (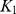), and completely independent of fluctuations in the signaling protein concentrations (cf. Figure 2B)
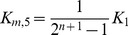
(10)


This protein concentration insensitivity can be explained as follows: strong negative feedback shifts the species 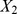 to 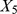 to very low activation levels, implying that the downstream part of the cascade does not saturate. The dose-response curve of the cascade thus follows the receptor-ligand binding isotherm, though with a more gradual shape. Such pathway alignment to the receptor dose-response curve due to negative feedback has been observed experimentally in the yeast pheromone signaling cascade [34].

The effects of strong negative feedback on the maximal pathway activation variability are less pronounced. Assuming log-normally distributed gene expression noise, the variance of 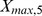 can be derived from Eq. 9 and represented as a function of the signaling protein concentration variances (Supplemental Text S1)
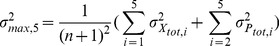
(11)


The 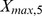 variability is determined by the sum of all protein concentration variances, but is reduced by the feedback term 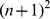. This result confirms previous observations showing that cooperative feedback 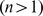 suppresses noise more efficiently than linear feedback [36].

The 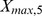 and 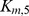 variabilities of the feedback system are independent of the activation resistances in the cascade and low compared to a feedback-less cascade (thin solid and thin dashed lines in Figure 2C). Thus, negative feedback allows for the simultaneous invariance of the maximal pathway activation and the pathway sensitivity, thereby resolving the robustness trade-off of the feedback-less cascade. Moreover, the negative feedback system shows the same signaling variability for low and high stimulus levels (Eq. 9), implying that quantitative information transmission is possible over a very broad stimulus concentration range. These conclusions continue to hold for an equivalent negative feedback system, where the terminal kinase 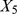 inhibits the activity of 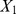, thereby controlling the phosphorylation reaction of 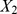. This can be seen in the steady state condition 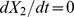 (Eq. 8) which can be converted to the kinase inhibition case by division with the feedback term 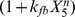.

We confirmed our findings concerning negative feedback regulation for more realistic feedback cascades with limited feedback strength 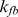. Figure 2C shows that the moderate feedback system shows a simultaneous invariance of maximal pathway activation (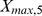) and the pathway sensitivity (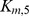) over a finite range of activation resistances in the cascade (thick solid lines), and the variability tends to be lower than that of a feedback-less cascade (dashed lines). The strength of the feedback regulation 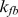 primarily affects the width of the compromise range where 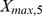 and 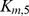 are simultaneously invariant: Limited feedback cannot perform any regulatory function for high activation resistances (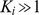), because 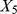 is barely activated in this regime. Likewise, moderate feedback cannot efficiently counteract the strong signaling activity of a cascade with too low activation resistance (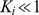).

#### 2.4 Negative feedback loops acting upstream and downstream in the cascade control different aspects of the dose-response curve

Signaling cascades are often equipped with multiple negative feedback loops, some acting close to the receptor level, while others modulate the terminal cascade levels [23]. We investigated how the length of a negative feedback emanating from 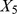 affects the dose-response behavior of the cascade. Consider a cascade with a short, downstream feedback, where 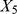 activates its own phosphatase (Figure 2A, dashed red line). Such downstream feedback regulation occurs in the yeast pheromone pathway, as Msg5, the phosphatase acting at the terminal cascade level, is transcriptionally induced upon stimulation [22,37]. Again, most of the ODEs remain unchanged when compared to the basic cascade model (Eq. 1), but the fifth pathway level reads:
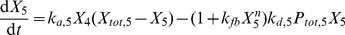
(12)


The steady state condition of the upstream feedback 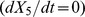 also describes an equivalent negative feedback system, where the terminal kinase 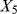 inhibits the activity of its own activator 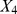. This can be seen by dividing the steady state condition with the feedback term 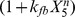. We again approximate the steady state for strong feedback (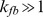) and obtain (Supplemental Text S1)
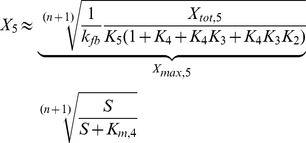
(13)


The steady state of 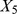 is proportional to the root of the Michaelis-Menten equation, and the dose-response curve is thus as shallow as that of the system with upstream feedback (Eq. 9). In similarity to Eq. 10, the half-saturation point of Eq. 13 is proportional to the half-maximal stimulus of 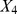 (i.e., 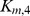). Downstream feedback thus eliminates the impact of the terminal level on the pathway sensitivity, but any variability arising between 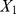 and 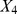 is transmitted. Downstream feedback suppresses the variability of the maximal pathway activation, especially for high feedback cooperativity n (Eq. 13), and achieves the same or stronger 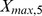 invariance when compared to the upstream feedback system (thin orange lines in Figure 2C and Figure 2D): this is because the upstream signaling protein concentrations (parameters 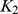, 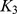 and 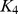) have a lower impact in Eq. 13 than in Eq. 9 if the phosphatase activities in the cascade are low 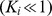. We conclude that only upstream feedback efficiently suppresses 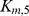 fluctuations, while downstream feedback has the stronger impact on the 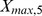 variability. For both systems, increasing feedback cooperativity 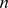 selectively suppresses the 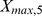 fluctuations.

We analyzed the downstream feedback model with limited feedback strength 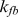 (thick lines in Figure 2D). Moderate downstream feedback also resolves the robustness trade-off in protein kinase signaling by allowing for a simultaneous 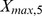 and 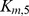 invariance at intermediate activation resistances. Moderate downstream feedback reduces 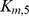 fluctuations to a much lesser extent than upstream feedback, while having a slightly more pronounced effect on the 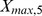 variability (Figure 2C and Figure 2D). Taken together, upstream and downstream feedback loops differentially control the dose-response behavior also at moderate feedback strengths, although the differences are less pronounced compared to the case of strong feedback (Figure 2C and Figure 2D).

Our models predict that upstream negative feedback in the pheromone pathway may contribute to the invariant shmooing threshold, while downstream negative feedbacks may primarily ensure that all cells exhibit a similar maximal activation upon strong stimulation. One limitation of the negative feedback models is their shallow dose-response behavior which is inconsistent with the reported ultrasensitivity of the pheromone pathway [18,20,21]. We turn to ultrasensitive signaling cascades in the following to study more realistic models of yeast pheromone sensing.

### 3 Cell-to-cell variability of ultrasensitive signaling cascades

The term ultrasensitivity describes signaling cascades with steep, sigmoidal dose-response curves that allow for all-or-none decision making. Ultrasensitive behavior has been reported for the yeast pheromone pathway, although the steepness of the dose-response curve differs between literature reports [18,20,21]. Various molecular mechanisms establish ultrasensitivity in signaling cascades, e.g., double phosphorylation or competitive inhibition [3,38]. In this work, we neglect the mechanistic details underlying ultrasensitive regulation, and represent ultrasensitivity at one or more cascade levels by the Hill equation (see below). This modeling approach allows us to study the propagation of variability in ultrasensitive signaling cascades.

Two strategies exist to establish a very steep overall dose-response curve in a signaling cascade: Firstly, the all-or-none behavior may be primarily established at a single level, while the rest of the cascade shows gradual behavior (in isolation). Localized switching at the terminal cascade level has been reported for the yeast mating pathway [18]. Secondly, switching may be distributed over multiple steps, i.e., each cascade level exhibits mild ultrasensitivity in isolation and cascade amplification effects ensure that the overall dose-response curve is very steep. Such behavior has been observed for the MAPK cascade in Xenopus oocytes [39], and is likely to be relevant for other MAPK cascades like the yeast pheromone pathway. The following discussion of cell-to-cell variability will initially focus on the second mode of distributed ultrasensitive decision making, before turning to the case of focused switching at a single level.

#### 3.1 Ultrasensitive cascades with distributed switching can be inherently invariant

Multi-step signaling cascades are capable of strong ultrasensitivity amplification, implying that a combination of multiple weak switches establishes a very steep overall dose-response curve. To simplify the mathematical analysis, we initially analyze a two-step signaling cascade with ultrasensitivity at each level
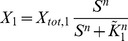
(14)

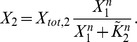
The steady state of each cascade level is represented by the Hill equation, which has a structure analogous to the local dose-response behavior of a gradual signaling cascade (Eq. 2). The maximal activation of each cascade level equals the total concentration of the respective kinase (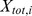), and the half-saturation point is determined by the parameters 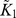 and 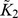. 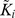 equals the equivalence point of kinase and phosphatase activities in ultrasensitive (de)phosphorylation systems, and is thus determined by the concentration of a phosphatase [5]. Ultrasensitive, sigmoidal dose-response behavior can be observed for Hill coefficients 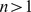.

We analyzed the overall dose-response curve relating the signaling output 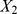 to the stimulus 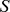, and found that the ultrasensitive behavior is amplified along the cascade (Supplemental Text S1). Assuming a low phosphatase activity at the second level (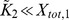), the threshold where the system switches from low to high activation is given by
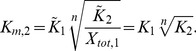
(15)


The activation resistances 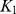 and 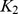 are defined in Eq. 5. The threshold depends in a less-than linear manner on the kinase and phosphatase concentrations controlling the second level (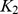), thus showing little variability. At the same time, the maximal activation level depends the concentration of the terminal kinase only (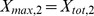) and shows partial invariance as well. The ultrasensitive system thus shows a less pronounced robustness trade-off when compared to the gradual system, and can simultaneously show little variability of 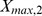 and 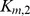.

**Figure 3:**
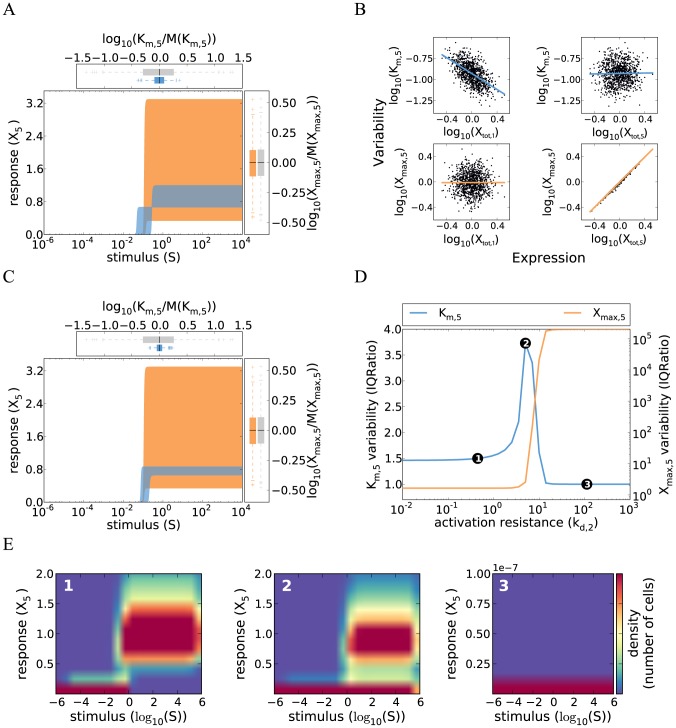
Cell-to-cell variability of kinase cascades with distributed ultrasensitive switching. **A** Simulations of a cascade with distributed ultrasensitive switching and low activation resistance shows a steep response with little variability in 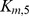 (defined as the stimulus for a half-maximal pathway activation). The simulations of the five-step cascade were performed by iteratively applying the Hill equation describing the steady state of each level (similar to Eq. 14). The concepts and parameter values correspond to Figure 1B, with a Hill coefficient 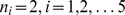 (Supplemental Table S1). Colored box plots represent the 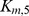 and 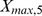 distribution of the ultrasensitive model, while gray box plots show the behavior of the reference gradual cascade (cf. Figure 1B). **B** 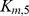 is strongly controlled by the first kinase concentration, whereas 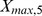 primarily responds to fluctuations in the terminal kinase (concept similar to Figure 1D). **C** Simulations of a cascade with distributed ultrasensitive switching show that the threshold variability can be reduced by coregulating the first level kinase (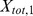) and second level phosphatase (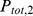) concentrations. Correlation was modeled by introducing a proportional relationship between both concentrations. **D**–**E** The variabilities of 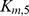 and 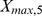 were analyzed using the IQRatio as in Figure 1C, but plotted against changes in the kinetic parameter value for only the second level phosphatase (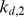). Similar results are obtained using the coefficient of variation as a measure of variability (Figure S3). The markers 1–3 correspond to the respective dose-response density plots shown in **E**. A high density (red) corresponds to a high number of cells showing a particular stimulus-response relationship. Three modes of variability are visible in **E**: 1) for low resistance values, the variability in 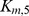 is low and all cells are able to respond to stimulation; 2) the variability increases at intermediate resistance levels, because only a fraction of the cells respond while the remaining cells do not even for high stimulus values; 3) in case of a high activation resistance no cell is able to respond.

Numerical simulations were performed for a five-step signaling cascade, where each level was modeled using a Hill equation with n = 2 (similar to Eq. 14). The five-step signaling cascade exhibits very strong ultrasensitivity if low phosphatase activities are assumed for all cascade levels (Figure 3A; 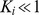). The system shows little cell-to-cell variability, as all cells respond in a switch-like manner within a ∼3-fold range of stimulus concentrations. As with the two-step cascade, the threshold in these cell-to-cell variability simulations is almost exclusively determined by the upstream signaling species: 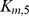 correlates well with the concentration of the first kinase 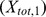, but not with signaling protein concentrations controlling subsequent cascade steps (Figure 3B).

The inherent invariance of the ultrasensitive system can be understood intuitively by considering an extreme case scenario, where each cascade level is a very steep switch (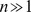): In this case, all downstream cascade levels simultaneously respond as soon as the first level is switched on. The system thus behaves like a chain of dominos, and the threshold of the first level sets the threshold of the whole cascade. The phenomenon is less pronounced for the case of moderate switching at each step, so that the downstream protein concentrations still matter to some extent (Eq. 15).

We conclude that the coordinated switching of the whole yeast cell population within a ∼3-fold range of pheromone concentrations could be explained based on the ultrasensitive model with distributed switching (Figure 3A). The prediction of local ultrasensitivity which is then amplified along the cascade could be tested experimentally by measuring and relating the dose-response curves of several kinases in the cascade.

#### 3.2 Active variability regulation in ultrasensitive cascades by gene expression noise control and parameter tuning

The threshold variability of the ultrasensitive system strongly depends on the noise of 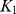, the kinase-phosphatase ratio at the upstream cascade level (Eq. 15; Figure 3B).The noise of 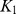 could be reduced by correlating the fluctuations of the respective kinase and phosphatase concentrations. Such correlated fluctuations may be realized in the yeast pheromone pathway, because the pheromone receptor Ste2 and the antagonizing G protein deactivator Sst2 are transcriptionally co-regulated by the transcription factor Ste12 [22,37]. In Figure 3C, we simulated the five-step signaling cascade with moderate switching at each level, and introduced correlated fluctuations between the first kinase concentration 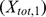 and the antagonizing second phosphatase concentration 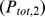. We find that this system exhibits less variability when compared to the uncorrelated case, as all cells respond in a switch-like manner within a ∼2-fold range of stimulus concentrations (compare Figure 3A and Figure 3C). Experimental work supports that correlated fluctuations in upstream kinase and phosphatase concentrations reduce the variability of mammalian MAPK signaling [7]. We propose to simultaneously measure the expression levels of fluorescently labeled Ste2 and Sst2 in single-cells to confirm that a similar mechanism promotes the invariance of yeast shmooing.

Correlated fluctuations in a single kinase-phosphatase pair would also promote invariance in gradual signaling cascades, but only to a minor extent, because the remaining seven protein concentration variabilities still enter the signaling activity upon weak stimulation in an additive manner (Eqs. 6 and 7): The cell-to-cell variability of a gradual cascade with realistic protein concentration fluctuations, quantified as the inter-quartile ratio (see Methods), only drops from 4.1 to 3.5 if a perfect correlation is introduced for a single kinase-phosphatase pair. This suggests that correlations in upstream signaling protein concentrations specifically promote the robustness of ultrasensitive systems.

A way to increase the variability of the ultrasensitive cascade relative to Figure 3A is kinetic parameter tuning, e.g., by increasing the activity of certain phosphatases. Figure 3D shows the variabilities of maximal pathway activation (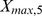) and the pathway sensitivity (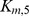) for varying phosphatase expression at the second level. Both variabilities clearly increase for increasing phosphatase expression, and the variance of 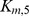 peaks at intermediate levels. Increasing phosphatase expression introduces heterogeneity because a fraction of the cell population becomes completely insensitive to stimulation. This can be seen in Figure 3E, where the dose-response curve distributions of the cell population are indicated by density plots for different phosphatase levels. For instance, at intermediate phosphatase levels, half of the cells do not respond at all to stimulation, while the remainder shows essentially complete activation of 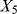 (Figure 3E, panel 2). Thus, increasing phosphatase expression introduces heterogeneity, because the system switches from a strong and synchronous response of the whole population to a strong response in only a fraction of cells. The 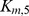 variability peaks at intermediate phosphatase activities, because the stimulus level required for half-maximal activation is different in responding and non-responding cells (not shown). At very high phosphatase levels, the population only consists of non-responders, thus again showing less variability (Figure 3E, panel 3).

#### 3.3 Ultrasensitive signaling cascades with switching at a single step show strong variability

Switch-like decision making may also be established if a single cascade level shows a very steep dose-response curve. Such a localized switch has been reported for the yeast mating pathway [18]: The scaffold protein Ste5 co-localizes members of the MAPK cascade, and its activity is regulated by a multisite dephosphorylation mechanism, thereby promoting the switch-like phosphorylation of the terminal MAPK cascade member Fus3. We mimic this scenario by assuming that the terminal cascade level 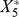 is phosphorylated by 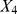 in a switch-like manner, whereas the upstream part of the pathway 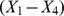 shows gradual behavior. In similarity to Eq. 3 the steady state of 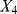 can be written as
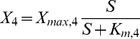
(16)


The switch-like dose-response at the terminal level (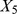) may be represented by the Hill equation :
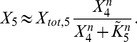
(17)


**Figure 4:**
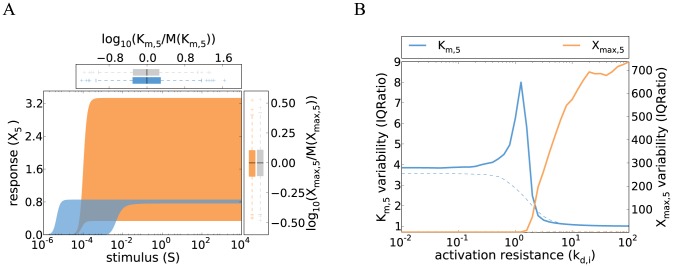
Cell-to-cell variability of cascades with a localized switch at the terminal level. **A** Simulations show that pronounced variability for both 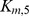 (defined as the stimulus for a half-maximal pathway activation) and 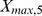. The concepts and parameter values correspond to Figure 1B, and the simulations were performed by iteratively applying Eqs. 16 and 17 with a Hill coefficient 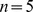 (Supplemental Table S1). Colored box plots represent the 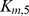 and 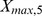 distribution of the ultrasensitive model, while gray box plots show the behavior of the reference gradual cascade (cf. Figure 1B). **B** The variabilities of a cascade with a localized switch at the terminal level were analyzed using the IQRatio, and the activation resistance was tuned by varying several phosphatase rate constants (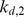
– 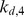, thick, solid lines), and compared to a gradual model (thin, dashed lines). In contrast to a cascade with distributed ultrasensitivity (Figure 3D), homogeneous switching of all cells at a defined stimulus value is not possible even for low activation resistances. Similar results are obtained using the coefficient of variation as a measure of variability (Figure S4).

The threshold 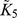 determines the equivalence point of kinase and phosphatase activities at the fifth level, and is proportional to the concentration of the phosphatase 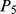. We performed cell-to-cell variability simulations of this system assuming a high Hill coefficient n (Figure 4A), and investigated how the variabilities of maximal pathway activation and threshold stimulus depend on the kinetic parameter values (Figure 4B). Increasing phosphatase expression shifts the system from complete switching of the whole cell population to incomplete switching of only a fraction of cells, reminiscent of the cascade with distributed switching (Figure 3E). Cells only respond to stimulation if the maximal activation level of 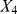 is larger than the Hill equation threshold 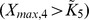. The single-switch system shows strong variability even for low phosphatase activities in the cascade, because seven signaling protein concentrations jointly determine the signaling threshold (Supplemental Text S1). The simulated signaling thresholds vary over three orders of magnitude as long as all cells of the population respond strongly to stimulation (Figure 4A), implying that the single switch system cannot explain the experimentally observed invariance of the shmooing threshold (see Rationale). We show in the following that invariance can be realized if the single-switch model is extended by feedback or feedforward loops.

#### 3.4 Suppression of signaling threshold variability by basal transcriptional feedback

Negative feedback diminishes the variability of signaling cascades, though at the cost of a reduced steepness of the dose-response curve (Eq. 9). In this Section, we demonstrate that switching and invariance can be combined if the time window of variability suppression by negative feedback can be separated from the time window of switch-like decision making.

Feedback loops in mammalian signaling commonly involve transcriptional regulation, and the signaling cascades typically induce the expression of their own inhibitors [23]. In the yeast mating pathway phosphatases negative regulators like the G protein deactivator Sst2 and the phosphatase Msg5 are transcriptionally induced upon stimulation [22,37]. Transcriptional feedback requires *de novo* protein biosynthesis, thereby affecting signal transduction with a delay. Thus, a time window exists early after stimulation where the steepness of the dose-response curve is unaffected by transcriptional feedback. Yet, transcriptional feedback may promote robustness of the dose-response curve if it operates under basal conditions before stimulation as observed for the yeast mating pathway [34,37,40]: Basal feedback inhibitors are able to correct for the basal state variability, because their concentration reflects and tunes the basal signaling activity. This variability suppression effect can be memorized to the time window of acute stimulation, as long as the concentration of the feedback inhibitor remains stable. In the following, it will be shown that this memorization of the pre-stimulation state efficiently suppresses variability upon acute stimulation.

**Figure 5:**
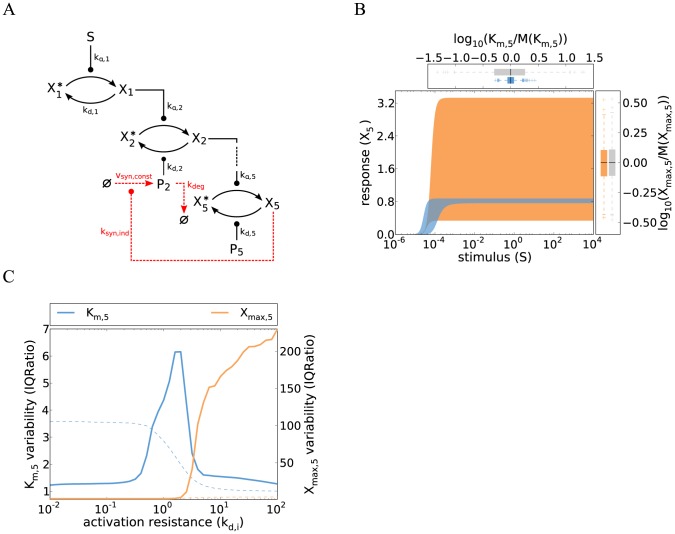
Cell-to-cell variability of cascades with a localized switch at the terminal level and basal transcriptional feedback. **A** Schematic representation of the five-step cascade with an ultrasensitive terminal step and basal transcriptional feedback. Assuming fast pathway dynamics and slow expression dynamics (time-scale separation), the system can be considered to exist in two states: at basal levels of stimulus, 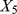 induces the expression of the second level phosphatase 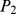 (Eq. 18). Upon acute stimulation the pathway responds rapidly but the expression kinetics of the phosphatase are too slow to establish a significant feedback regulation (Eq. 19). **B** Simulations of a cascade with a localized switch at the terminal level and basal transcriptional feedback show a reduced variability when compared to the ultrasensitive model without basal transcriptional feedback shown in Figure 4A. The concepts and parameter values correspond to Figure 1B, and the simulations were performed numerically integrating the ODE system given by Eqs. 16–19, with a Hill coefficient 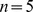, a basal stimulus of 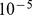, a basal synthesis rate 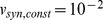, an 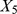-induced synthesis rate constant 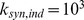, and a degradation rate 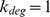 (Supplemental Table S1). Colored box plots represent the 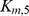 and 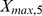 distribution of the basal transcriptional feedback model, while gray box plots show the behavior of the reference gradual cascade (cf. Figure 1B). **C** Variabilities of 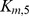 (defined as the stimulus for a half-maximal pathway activation) and 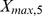 were analyzed using the IQRatio, and the activation resistance was tuned by varying several phosphatase rate constants (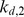
– 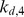, thick, solid lines). The variability of the gradual model is shown for comparison (thin, dashed lines). The variant with basal transcriptional feedback is able to strongly reduce the variability in 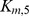 for low activation resistance values when compared to the single-switch model without feedback (cf. Figure 4B). Similar results are obtained using the coefficient of variation as a measure of variability (Figure S5).

We model basal transcriptional feedback by implementing a negative feedback loop in the single-switch cascade. The phosphatase at the second level is transcriptionally induced by the active terminal kinase 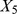 (Figure 5A) and also transcribed with a basal rate 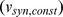. Additionally, 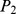 is subject to degradation 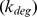, giving rise to the following differential equation
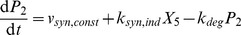
(18)


Basal signaling was implemented by assuming a low chronic level of the stimulus 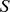, and ligand-induced signaling was simulated by further increasing 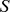. Time scale separation was introduced by neglecting the induction of 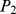 by transcriptional feedback early after stimulation. The concentration of 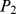 is thus fixed to the basal level throughout the time window of acute stimulation, i.e.,
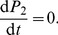
(19)


We performed cell-to-cell variability simulations of this system assuming a high Hill coefficient 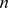 (Figure 5B). The basal transcriptional feedback model shows a strongly reduced threshold variability when compared to a feedback-less cascade with the same kinetic parameters (compare Figure 4A and Figure 5B). The simulated signaling thresholds lie within a ∼3-fold range of stimulus concentrations (Figure 5B), which is consistent with the experimentally observed invariance of the shmooing threshold (see Rationale). Strong variability suppression is possible, because the basal signaling activity and the pathway threshold are controlled by the same combination of parameters (Supplemental Text S1): Transcriptional feedbacks correcting for fluctuations in basal signaling thereby indirectly correct the threshold variability as well. The threshold invariance of the basal feedback system is more pronounced if the dose-response curve of the signaling pathway is highly switch-like, because cooperativity promotes robustness in negative feedback circuits.

The invariance due to basal feedback is restricted to a certain range of activation resistances in the cascade, because increasing the expression of several phosphatases in the cascade shifts the system from complete switching of the whole cell population to heterogeneous switching of only a fraction of cells (Figure 5C). In Figure 5B and Figure 5C, we assumed that heterogeneity in 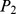 expression solely arises from fluctuations in the upstream signaling cascade. However, basal transcriptional feedback can exert a similar variability suppression if the 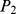 synthesis rate is also noisy and sampled from a log-normal distribution (not shown).

Recent experimental evidence supports that Msg5, a basal transcriptional feedback regulator of the yeast mating pathway, suppresses the signaling variability upon pheromone stimulation [26]. We propose to eliminate basal transcriptional feedback by exchanging the endogenous promoter of the Msg5 gene by a promoter that is not regulated by the pheromone, and predict a strong increase in the signaling threshold variability.

#### 3.5 Variability suppression by coherent feedforward regulation

Signaling networks commonly involve coherent feedforward loops where an upstream kinase controls a downstream target by two parallel pathways. Fus3, the yeast MAPK that mediates the shmooing response, is controlled by two pheromone-dependent pathways, both of which are required for full activation [18]: Fus3 is phosphorylated by the upstream MAP kinase kinase Ste7, and additionally needs to be released from an inhibitory site in the scaffold protein Ste5 for activation. The latter step involves Ste5 multisite dephosphorylation by the phosphatase Ptc1. It will be shown below that coherent feedforward regulation of Fus3 may contribute to the invariance of shmooing.

**Figure 6:**
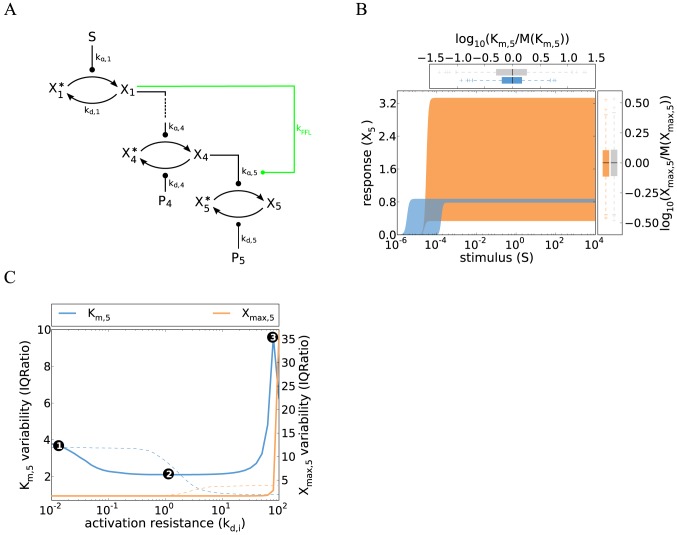
Cell-to-cell variability of cascades with coherent feedforward regulation. **A** Schematic representation of the five-step cascade with a coherent feedforward loop: the 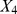-mediated phosphorylation of 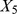 is positively regulated by the kinase 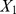 (see main text). **B** Simulations of a cascade with a coherent feedforward loop show reduced variability when compared to the single-switch model without feedforward regulation (Figure 4A). The concepts and parameter values correspond to Figure 1B, and the simulations were performed by iteratively applying Eqs. 16 and 20, with a Hill coefficient 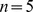 and 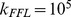 (see Supplemental Table S1). Colored box plots represent the 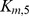 and 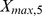 distribution of the feedforward model, while gray box plots show the behavior of the reference gradual cascade (cf. Figure 1B). **C** The variabilities of 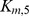 (defined as the stimulus for a half-maximal pathway activation) and 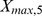 were analyzed as a function of the activation resistance by varying several phosphatase rate constants (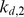
–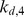, thick, solid lines), and compared to a gradual model (thin dashed lines). Feedforward regulation plays no role at low activation resistances (point 1), but reduces the variability at intermediate activation resistances (point 2; see main text). High variability arises at high resistances, because not all cells reach the threshold for full 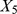 activation (point 3). Similar results are obtained using the coefficient of variation as a measure of variability (Figure S6).

We model coherent feedforward regulation by extending the model of the five-step signaling cascade with a switch at the terminal level (Eqs. 16 and 17). The downstream signaling species 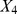 and 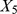 represent the MAPKK Ste7 and its target Fus3, respectively. Ptc1 is regulated by pheromone pathway intermediates upstream of Fus3 [18], but the precise molecular mechanism is not known. We assume in the model that the Ptc1 activity is directly activated by the pheromone receptor (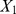). and that the Ptc1 pathway enhances 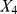
–mediated phosphorylation of 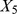 (Figure 6A). The effective kinase concentration is thus enhanced by the feedforward term 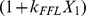 and the modified steady state equation for 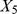 reads
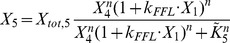
(20)


The threshold 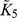 is proportional to the concentration of the phosphatase 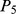, and thus subject to fluctuations. The parameter 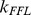 determines how strongly the phosphorylation of 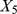 is enhanced by feedforward regulation. We assume strong crosstalk (large 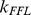) and neglect saturation in the upstream part of the cascade (i.e., 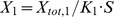 and 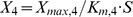) to ensure that both feedforward branches jointly regulate 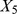 in a stimulus-dependent manner. The latter assumption is justified if the activation resistances in the cascade are large (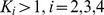). Then, steady state modifies to
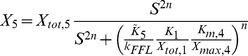
(21)


In line with previous reports, we find that coherent feedforward regulation increases the ultrasensitivity of the dose-response curve, as the stimulus now enters with the exponent 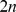 [41]. The switching threshold
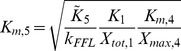
(22)contains the variabilities of 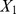 and 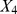 (determined by the ratios 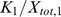 and 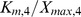, respectively). The total variability of the feedforward system is determined by the product of the individual branch variabilities, but each branch variability enters as a root only. Feedforward regulation thus reduces the threshold variability compared to the simple cascade if the feedforward branch is a shortcut and regulated by only a few signaling protein concentrations.

Explicit cell-to-cell variability simulations using Eq. 20 confirm that the threshold variability of the feedforward pathway (Figure 6B) is less than that of a simple ultrasensitive cascade with the same kinetic parameters (Figure 4A). Feedforward regulation robustly reduces the cell-to-cell variability, because partial invariance is observed over a broad range of activation resistances 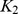
–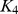 in the upstream cascade (point 2 in Figure 6C). The cell-to-cell variability increases for low activation resistances (point 1 in Figure 6C), because 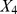 is activated at much lower stimulus levels than 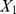, which prevents efficient feedforward regulation. For too high activation resistances, the maximal activation levels of 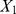 and 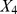 are too low to reliably activate 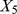, and the system shows high variability due to bimodal splitting into responding and non-responding cells (point 3 in Figure 6C).

We conclude that joint regulation of Fus3 by Ste7 and Ptc1 may reduce the cell-to-cell variability of pheromone signaling. However, the simulated signaling thresholds still span a ∼50-fold range of stimulus concentrations (Figure 6B), implying that feedforward regulation requires cooperation with other variability suppression mechanisms to bring about full robustness. The yeast pheromone pathway comprises feedforward loops other than the one comprising Ptc1, as G proteins employ several parallel pathways to activate the MAPK cascade [22]; it is possible that these multiple feedforward loops synergize to establish an invariant shmooing response.

## Discussion

A key question in biology is how cellular systems function robustly in face of internal and external fluctuations. We comprehensively characterized the determinants of cell-to-cell variability in protein kinase signaling cascades, and summarized our main findings in Table 1. Our work extends previous studies on cell-to-cell variability [6,42,43] and on variability reduction by negative feedback [9,33,44], bifunctional enzymes [45–47] or correlated protein concentration fluctuations [7,9,28,48]. We analyzed the steady state dose-response behavior of signaling systems, and showed that protein kinase cascades can be highly variable or inherently invariant, depending on the properties of individual reaction steps and their kinetic parameters. Our results may explain why the yeast pheromone pathway shows switch-like decision making with very little cell-to-cell variability.

**Table 1:** Determinants of cell-to-cell variability in gradual and ultrasensitive signal transduction.

Model		Dose-response behavior	Cell-to-cell variability	Note
Gradual	Eq. 1	gradual	high	variability most pronounced for weak stimulation
Feedback (upstream)	Eq. 8	very gradual	low	feedback primarily reduces pathway sensitivity (*K_m_*_,5_) fluctuations
Feedback (downstream)	Eq. 12	very gradual	low	feedback primarily reduces maximal pathway activation (*X_max_*_,5_) fluctuations
Distributed ultrasensitivity	Eq. 14	switch	low-high	variability can be regulated by phosphatase expression
Single-step ultrasensitivity	Eqs. 16, 17	switch	high	pathway threshold always highly variable
Single-step ultrasensitivity + basal transcriptional feedback	Eqs. 16–19	switch	low	switch-like decision making only for slow feedback possible
Single-step ultrasensitivity + coherent feedforward loop	Eqs. 16, 20	switch	medium	total variability is the combination of the individual branch variabilities

In this paper, we made a central simplifying assumption to study the behavior of protein kinase cascades: it was assumed that the individual levels of a protein kinase cascade function as isolated modules. Based on this assumption, we described the local dose-response behavior of each cascade level by Michaelis-Menten or Hill equations (Eqs. 3 and 14), and studied their behavior in tandem to gain insights into the global dose-response behavior of the five-step cascade. Depending on the protein concentrations and kinetic parameters in the cascade, the modularity assumption may be violated, and explicit simulations of all enzyme-substrate binding and dissociation events in the cascade may be necessary: strong sequestration of upstream kinases by highly abundant downstream substrates affects the phosphorylation state of the upstream kinase, thereby leading to retroactivity in the cascade [49–51]. Retroactivity results in positive or negative feedback regulation [49–51], and may therefore increase or decrease the cell-to-cell variability of protein kinase signaling. Sequestration effects and retroactivity can give rise to complex dynamic phenomena such as bistability and oscillations in computational models of MAPK signaling without explicit feedback regulation [52–55]. The cell-to-cell variability of such complex protein kinase signaling systems cannot be understood by analytical approaches, and thus needs to be analyzed numerically using extensive parameter sampling strategies [54]. Throughout this paper, we assumed that the signaling activity at each cascade level scales with the total kinase concentration (Eqs. 2, 14 and 17). However, a nonlinear relationship between signaling activity and total protein concentration is possible for (de)phosphorylation cycles with tight enzyme-substrate binding and sequestration effects [56,57], implying that the cell-to-cell variability would be increased or decreased.

Negative feedback is known to suppress the variability of biological systems and to reduce the steepness of signaling dose-response curves [32,33]. Here, we define more precisely the role of negative feedback in the modulation of signal transduction variability. Negative feedback simultaneously reduces the variability of the maximal pathway activation and the signaling threshold, thereby resolving the robustness trade-off which we observed in non-feedback cascades. The topological organization of the feedback loop determines which dose-response features are primarily affected by negative feedback: A feedback that acts upstream in the cascade primarily promotes invariance of the pathway threshold, while a feedback acting downstream controls the variability of the maximal pathway activation. We further find that the time scale of negative feedback regulation may determine its functional role: Variability suppression in fast, post-translational loops comes at the cost of a very shallow dose-response curve, implying that switch-like decision making is not possible. This trade-off can be circumvented in slow transcriptional feedback loops because the time windows of variability suppression and switch-like decision making can be separated. Interestingly, our simulations reveal that negative feedback acting upstream in signaling cascade may increase the cell-to-cell variability: For low phosphatase activities in the cascade (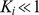), the maximal activation level of a feedback-less gradual cascade is determined by the terminal kinase concentration only and shows partial invariance (dashed orange line in Figure 2C). In contrast, the maximal activation level of a system with strong feedback is determined by multiple protein concentrations (Eq. 9), implying that negative feedback regulation increases the variability (solid orange line in Figure 2C).

In this paper, we analyzed the steady state behavior of negative feedback circuits, but did not focus on their dynamical properties such as sustained oscillations [58]. Interestingly, oscillations have been observed experimentally for yeast and mammalian MAPK cascades, and appear to be important for adequate cellular decision making [59,60]. We performed linear stability analyses to investigate whether sustained oscillations arise in our simple models of protein kinase signaling (Supplemental Text S1, Supplemental Figure S7 and Figure S8). As expected, oscillatory behavior was not possible in a simple multistep signaling cascade without negative feedback regulation (Eq. 1). Sustained oscillations were also not observed when the gradual kinase model was extended by a downstream negative feedback loop (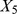 activates its own phosphatase; Eq. 12), because oscillations require a negative feedback with sufficient delay [58]. However, sustained oscillations can occur within a certain stimulus range in the model with upstream feedback (Eq. 8), provided that 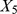 activates the phosphatase 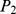 with high cooperativity 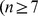). Such high feedback cooperativity is required to overcome saturation effects in the kinase cascade which compromise the emergence of sustained oscillations [58]. Damped oscillations already occur at lower feedback cooperativities. Our cell-to-cell variability simulations for the upstream feedback system thus represent the steady state behavior reached without damped oscillations (low 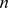), after damped oscillations (intermediate 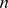) or the mean activity of a sustained oscillator (high 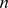).

One way to reduce noise in biological signaling systems is to correlate the expression fluctuations of antagonizing enzymes [7,9,28,48], e.g., by co-regulation at the transcriptional and/or post-transcriptional levels [61,62]. Our results indicate that efficient variability reduction by a correlated fluctuation of only two enzyme concentrations can only be achieved in ultrasensitive signaling pathways (Figure 3C). Gradual signaling systems require correlated fluctuations in most if not all signaling protein concentrations. Single-cell studies indicate that protein concentrations in the cell may be globally correlated, possibly due to fluctuations in RNA polymerase and/or ribosome copy numbers [12,28]. In our cell-to-cell variability simulations, we made a conservative assumption and neglected these protein concentration correlations. It is straightforward to extend our analytical results to the case of correlated fluctuations in all enzymes. Interestingly, several growth factor signaling pathways are organized in so-called synexpression groups, where most positive and negative regulators of signaling show tight spatio-temporal co-regulation [62]. Functional organization in synexpression groups may reflect the need for correlated fluctuations in multiple kinase-phosphatase pairs to effectively reduce variability. Most synexpression groups are transcriptionally controlled by the activity of their own signaling pathway, and thus combine multiple positive and negative transcriptional feedback loops. We find that signaling cascades with synexpression of multiple feedback regulators show little cell-to-cell variability, much like systems with co-expression of non-feedback regulators (unpublished observation).

Gradual signaling systems transduce information quantitatively and faithfully report the stimulus concentration in the extracellular milieu. We therefore assumed that the maximal pathway activation and the pathway sensitivity of a gradual system should be invariant. However, recent experimental work revealed that the absolute signaling activities of a mammalian MAPK cascade pathway are highly variable, while the stimulus-induced fold-change in the signal is invariant between cells [11]. Our results indicate that robust fold-change encoding is possible in a gradual signaling cascade with low phosphatase activities 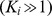: In this scenario, the pathway sensitivity is completely invariant (Eq. 4), and all cells show the same fold-change in response to a stimulus increase from one level to another. Future studies are required to investigate in more detail such alternative modes of robust signal transmission, especially in more complex models of protein kinase cascades.

## Materials and Methods

All simulations were done in Python. Analytical solutions were obtained using the open-source python package SymPy (www.sympy.org). Numerical simulations were performed using the odeint function of the scipy.integrate package (www.scipy.org). The details of the model implementation process can be found in the figure captions and in the Supplemental Protocol S1. The model parameters are listed in Supplemental Table S1. Source codes are available upon request.

Cell-to-cell variability was introduced into deterministic ordinary differential equation models of protein kinase signaling by assuming fluctuations in initial protein concentrations. The total kinase and phosphatase concentrations (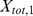, 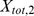, 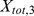, 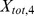, 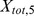, 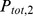, 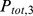, 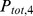, 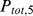) for each cell were sampled from independent log-normal distributions with a mean of 0 and a standard deviation of 0.35. The same kinetic parameter values were assumed for each cell of the population, and 1000 cells with different total protein concentrations were simulated for each model variant. The simulations of all model variants were performed with the same random number generator seed.

The steady state dose-response behavior of each cell was characterized by calculating the pathway sensitivity (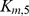) and the maximal pathway activation upon strong stimulation (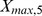). 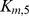 was calculated as the stimulus level leading to half-maximal pathway activation by finding the zero of the dose-response curve having a negative offset of 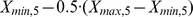, where 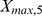 is the basal activation level in the absence of stimulation.

Various methods were employed to characterize the cell-to-cell variability of the signaling dose-response curves. In Figures 1B, 2B, 3A, 3C, 4A, 5B, and 6B, we explicitly show simulations of the dose-response behavior for specific parameter configurations, and highlighted cells with the highest and lowest 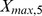 or 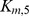 by the shaded areas (orange and blue, respectively). Since these population outliers could be subject to randomness, we additionally provide box plots at the top and the right to characterize the (normalized) 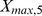 and 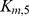 distributions, respectively (see Figures 1B, 2B, 3A, 3C, 4A, 5B, and 6B). The cell-to-cell variability for different parameter configurations was characterized using the interquartile (IQ) ratio (Figures 1C, 2C, 2D, 3D, 4B, 5C, and 6C), which was calculated as the ratio of the third (Q3) and first (Q1) quartile using the Python package NumPy (www.numpy.org). The IQ Ratio is a dimensionless number that reflects the fold difference between cells with high and low levels, while excluding (extreme) outliers. To further support our findings, we show in the Supplemental Figures S1, S2, S3, S4, S5, S6 that very similar results are obtained when using the coefficient of variation (CV = mean/standard deviation) as a measure of variability.

## Supporting Information

Figure S1
**Cell-to-cell variability of a gradual kinase cascade quantified using the coefficient of variation.** Concepts similar to Figure 1C, but the variabilities of 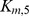 and 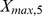 were analyzed using the coefficient of variation (CV = standard deviation/mean). High CVs imply high cell-to-cell variability, while 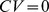 corresponds to no variability.(PDF)

Figure S2
**Cell-to-cell variability of gradual kinase cascades with negative feedback regulation quantified using the coefficient of variation.** The concepts in panels A and B are similar to Figure 2C and Figure 2D, respectively: The variabilities of 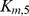 and 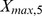 were analyzed using the coefficient of variation (CV = standard deviation/mean). The behavior of a feedback model with limited feedback strength (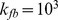 ; thick, solid lines) is compared to a feedback-less model (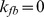; thin, dashed lines) and to a model with very strong feedback 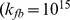; thin, solid lines).(PDF)

Figure S3
**Cell-to-cell variability of ultrasensitive kinase cascades with distributed switching quantified using the coefficient of variation.** The concepts are similar to Figure 3D, but the variabilities of 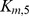 and 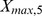 were analyzed using the coefficient of variation (CV = standard deviation/mean).(PDF)

Figure S4
**Cell-to-cell variability of kinase cascades with ultrasensitive switching at a single step quantified using the coefficient of variation.** The concepts are similar to Figure 4B, but the variabilities of 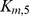 and 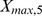 were analyzed using the coefficient of variation (CV = standard deviation/mean) and compared to the gradual model (thin, dashed lines).(PDF)

Figure S5
**Cell-to-cell variability of ultrasensitive kinase cascades with basal transcriptional feedback quantified using the coefficient of variation.** The concepts are similar to Figure 5C (main text), but the variabilities of 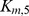 and 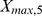 were analyzed using the coefficient of variation (CV = standard deviation/mean) and compared to the gradual model (thin, dashed lines).(PDF)

Figure S6
**Cell-to-cell variability of ultrasensitive kinase cascades with feedforward regulation quantified using the coefficient of variation.** The concepts are similar to Figure 6C, but the variabilities of 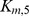 and 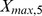 were analyzed using the coefficient of variation (CV = standard deviation/mean) and compared to the gradual model (thin, dashed lines).(PDF)

Figure S7
**Numerical stability analysis of the upstream feedback cascade with low activation resistances reveals that high feedback cooperativity is required for sustained oscillations.** The eigenvalues of the Jacobi matrix at the steady state were calculated numerically for different feedback cooperativities 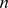 and stimuli 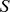 as described in Supplemental Text S1. The upper panel shows a classification of the real parts of the eigenvalues (blue: real parts of all eigenvalues are negative; red: real part of at least one eigenvalue is zero or positive). The lower panel indicates whether at least one of the eigenvalues is complex (red regions) or not (blue regions). Oscillations require that at least one of the eigenvalues is complex (red regions, lower panel). A damped oscillator exhibits only negative real parts (blue regions, upper panel), while at least one real part is zero or positive for sustained oscillators (red regions, upper panel). Sustained oscillations require very strong feedback cooperativity, (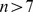). All activation resistances in the cascade were assumed to be low 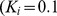, Eq. 5, main text). The simulations cover the full dynamic range of the dose-response curves. Parameters: 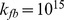, 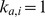, 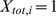 and 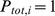, 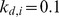.(PDF)

Figure S8
**Numerical stability analysis of the upstream feedback cascade with high activation resistances reveals that sustained oscillations are not possible even for very strong feedback cooperativity.** The concepts are similar to Figure S7, but all activation resistances in the cascade were assumed to be high (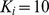, Eq. 5, main text). Parameters: 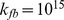, 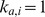, 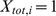 and 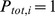, 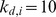.(PDF)

Protocol S1 **Implementation of model variants.**
(PDF)

Table S1
**Parameter values used for simulations.** The index 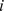 runs over the set 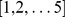 if not specified explicitely.(PDF)

Text S1 **Analytical derivations and numerical analysis of steady state stability.**
(PDF)
