# Supplementary figures and images for: Determinants of Cell-to-Cell Variability in Protein Kinase Signaling
Source: PLoS Comput Biol. 2013 Dec 5;9(12):e1003357. doi: 10.1371/journal.pcbi.1003357 (PMC3854479; doi:10.1371/journal.pcbi.1003357)

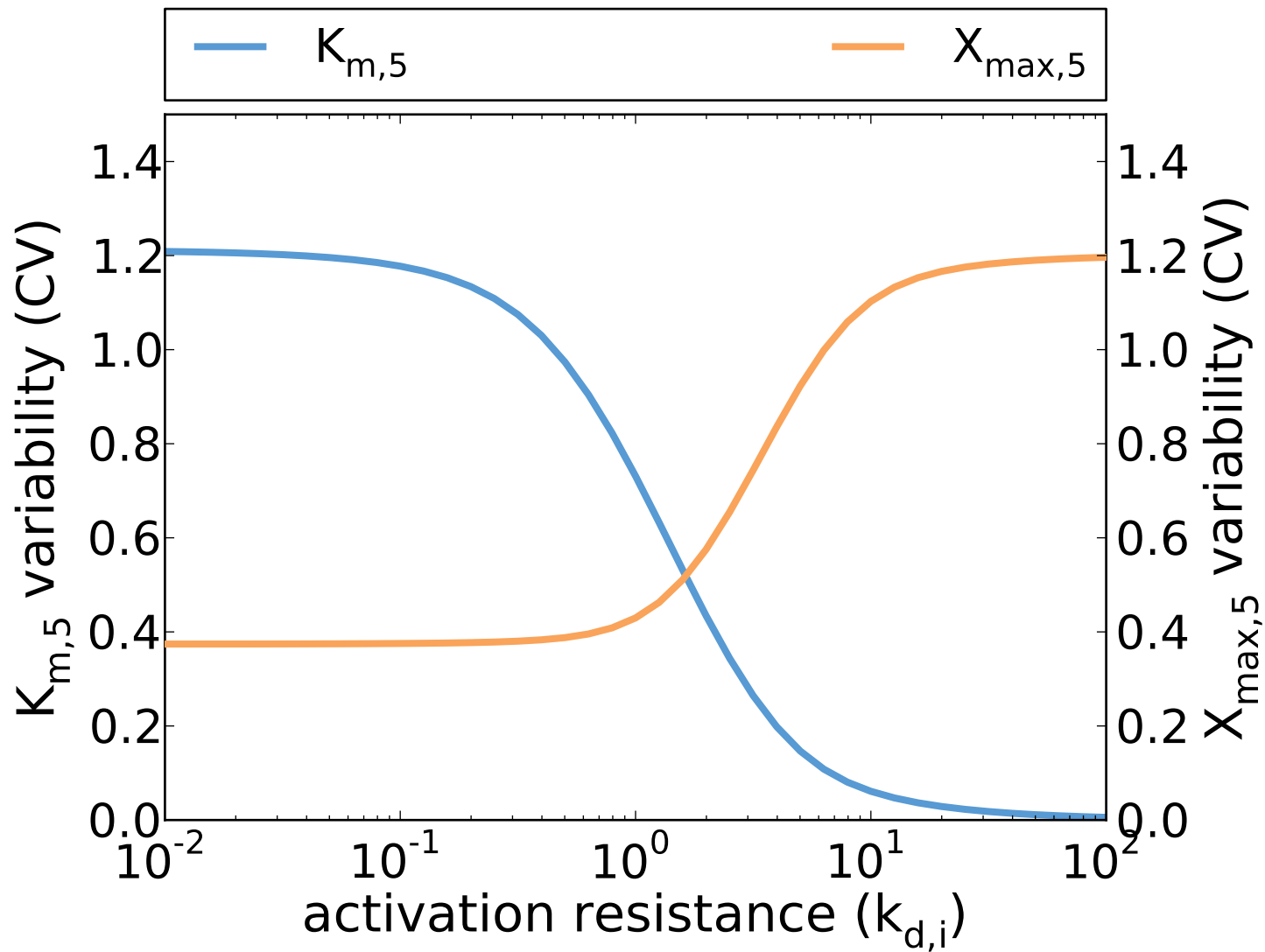

Supplement: Figure S1 — Cell-to-cell variability of a gradual kinase cascade quantified using the coefficient of variation. Concepts similar to Figure 1C, but the variabilities of and were analyzed using the coefficient of variation (CV = standard deviation/mean). High CVs imply high cell-to-cell variability, while corresponds to no variability. (PDF) [file pcbi.1003357.s001.pdf]

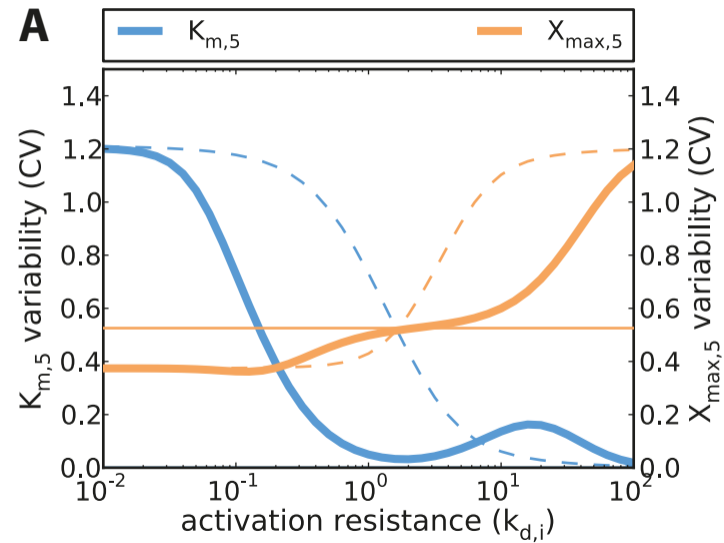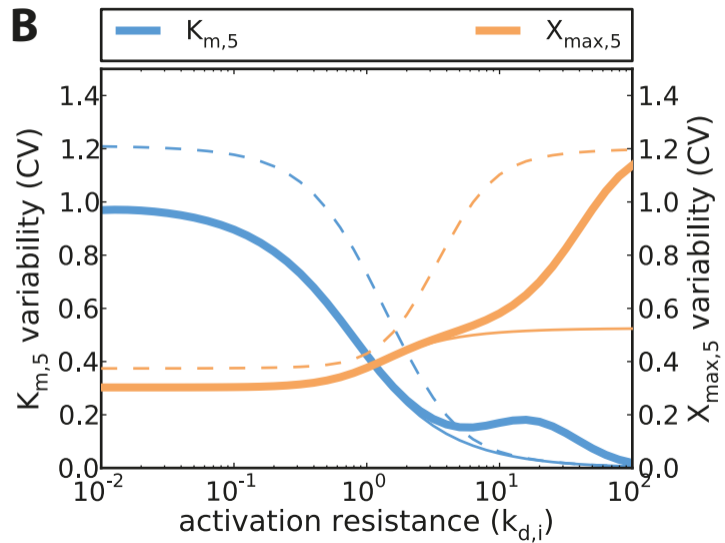

Supplement: Figure S2 — Cell-to-cell variability of gradual kinase cascades with negative feedback regulation quantified using the coefficient of variation. The concepts in panels A and B are similar to Figure 2C and Figure 2D, respectively: The variabilities of and were analyzed using the coefficient of variation (CV = standard deviation/mean). The behavior of a feedback model with limited feedback strength ( ; thick, solid lines) is compared to a feedback-less model (; thin, dashed lines) and to a model with very strong feedback ; thin, solid lines). (PDF) [file pcbi.1003357.s002.pdf]

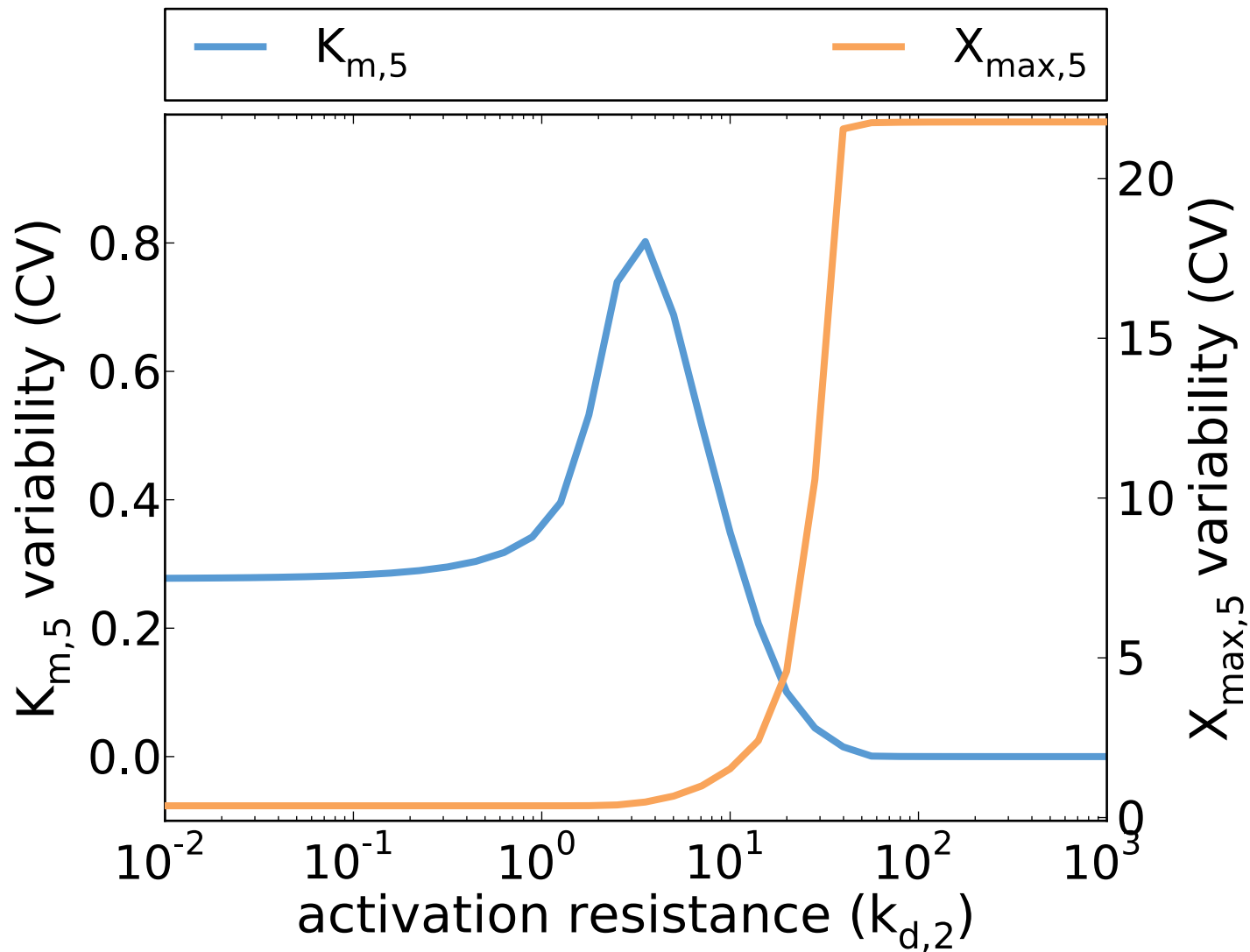

Supplement: Figure S3 — Cell-to-cell variability of ultrasensitive kinase cascades with distributed switching quantified using the coefficient of variation. The concepts are similar to Figure 3D, but the variabilities of and were analyzed using the coefficient of variation (CV = standard deviation/mean). (PDF) [file pcbi.1003357.s003.pdf]

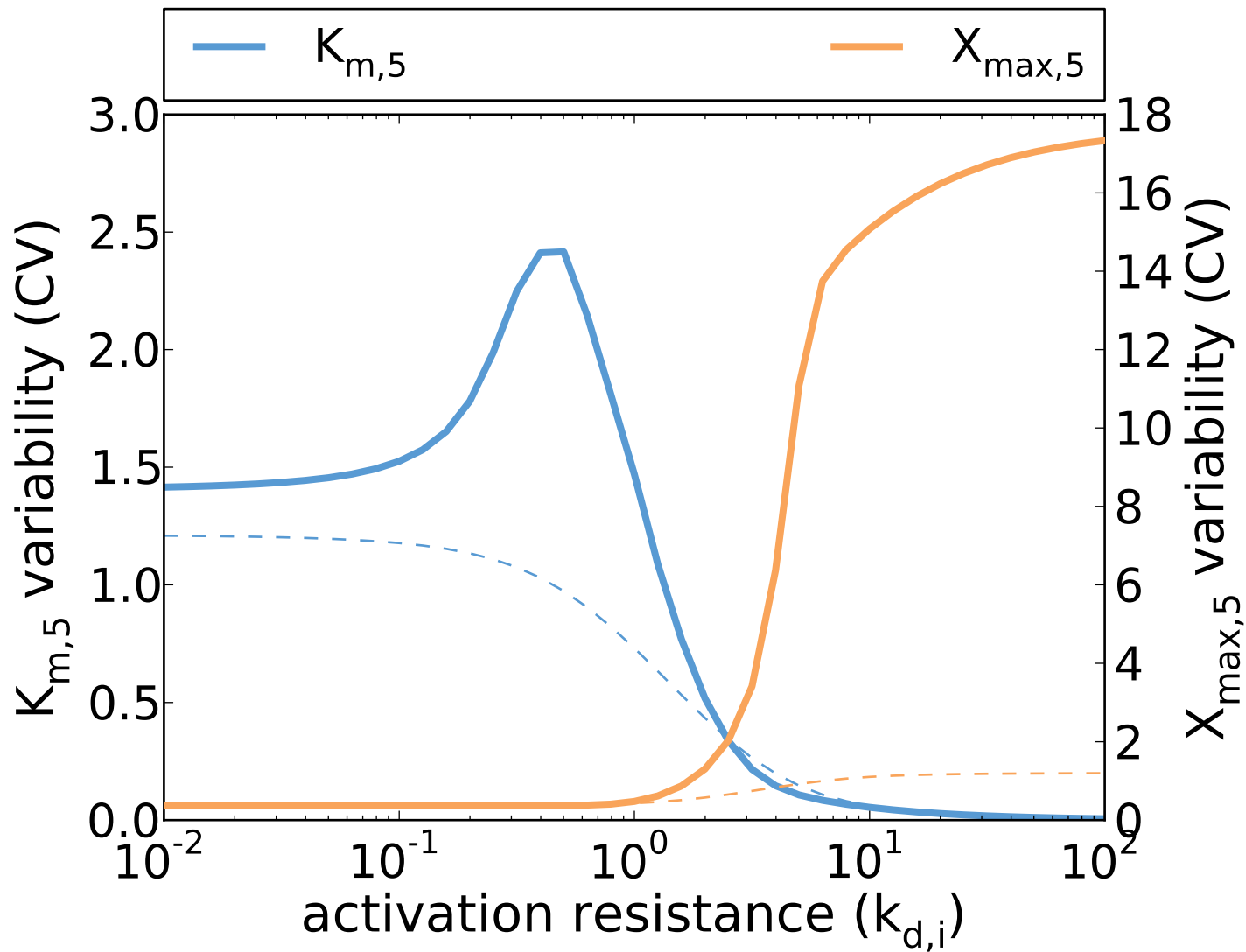

Supplement: Figure S4 — Cell-to-cell variability of kinase cascades with ultrasensitive switching at a single step quantified using the coefficient of variation. The concepts are similar to Figure 4B, but the variabilities of and were analyzed using the coefficient of variation (CV = standard deviation/mean) and compared to the gradual model (thin, dashed lines). (PDF) [file pcbi.1003357.s004.pdf]

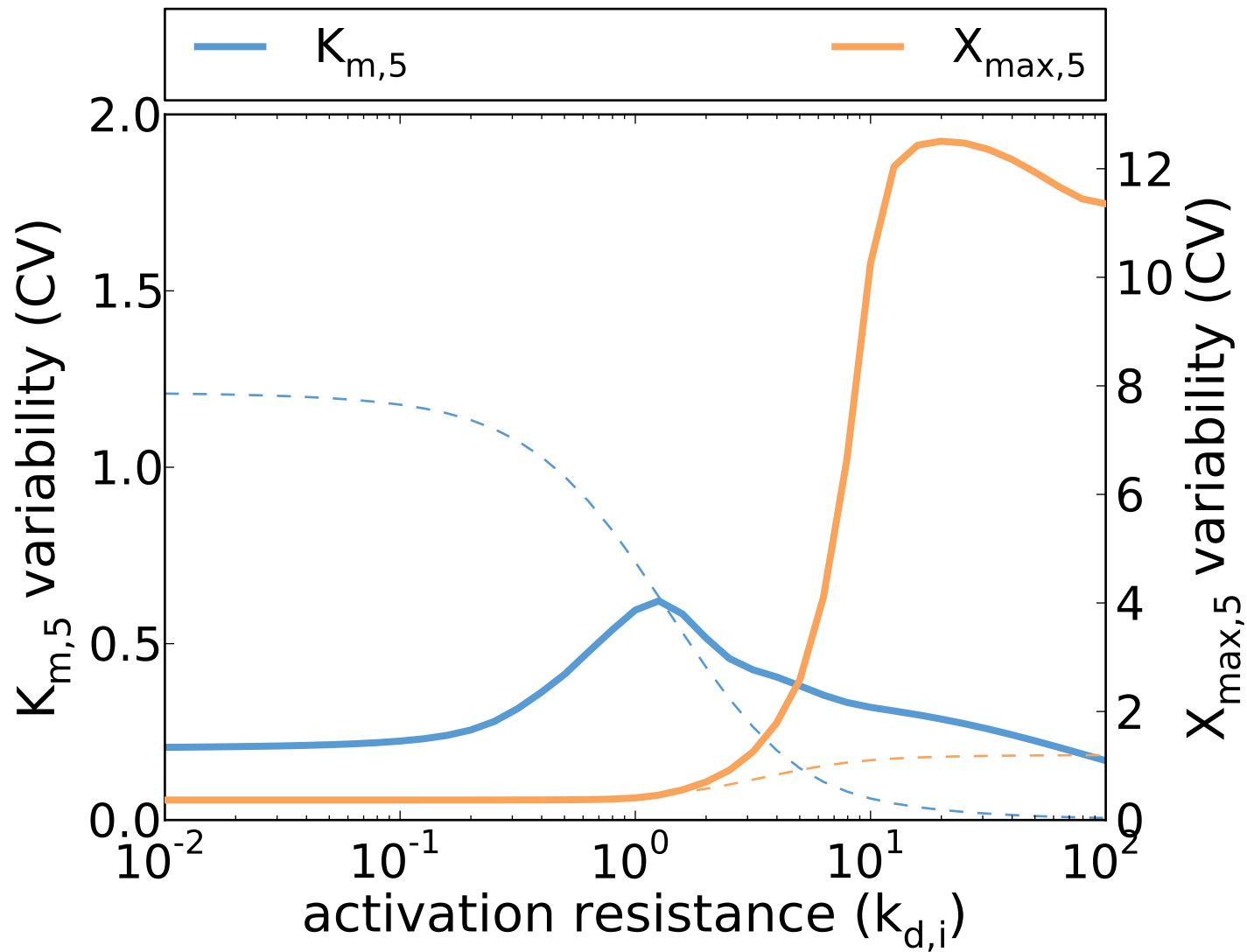

Supplement: Figure S5 — Cell-to-cell variability of ultrasensitive kinase cascades with basal transcriptional feedback quantified using the coefficient of variation. The concepts are similar to Figure 5C (main text), but the variabilities of and were analyzed using the coefficient of variation (CV = standard deviation/mean) and compared to the gradual model (thin, dashed lines). (PDF) [file pcbi.1003357.s005.pdf]

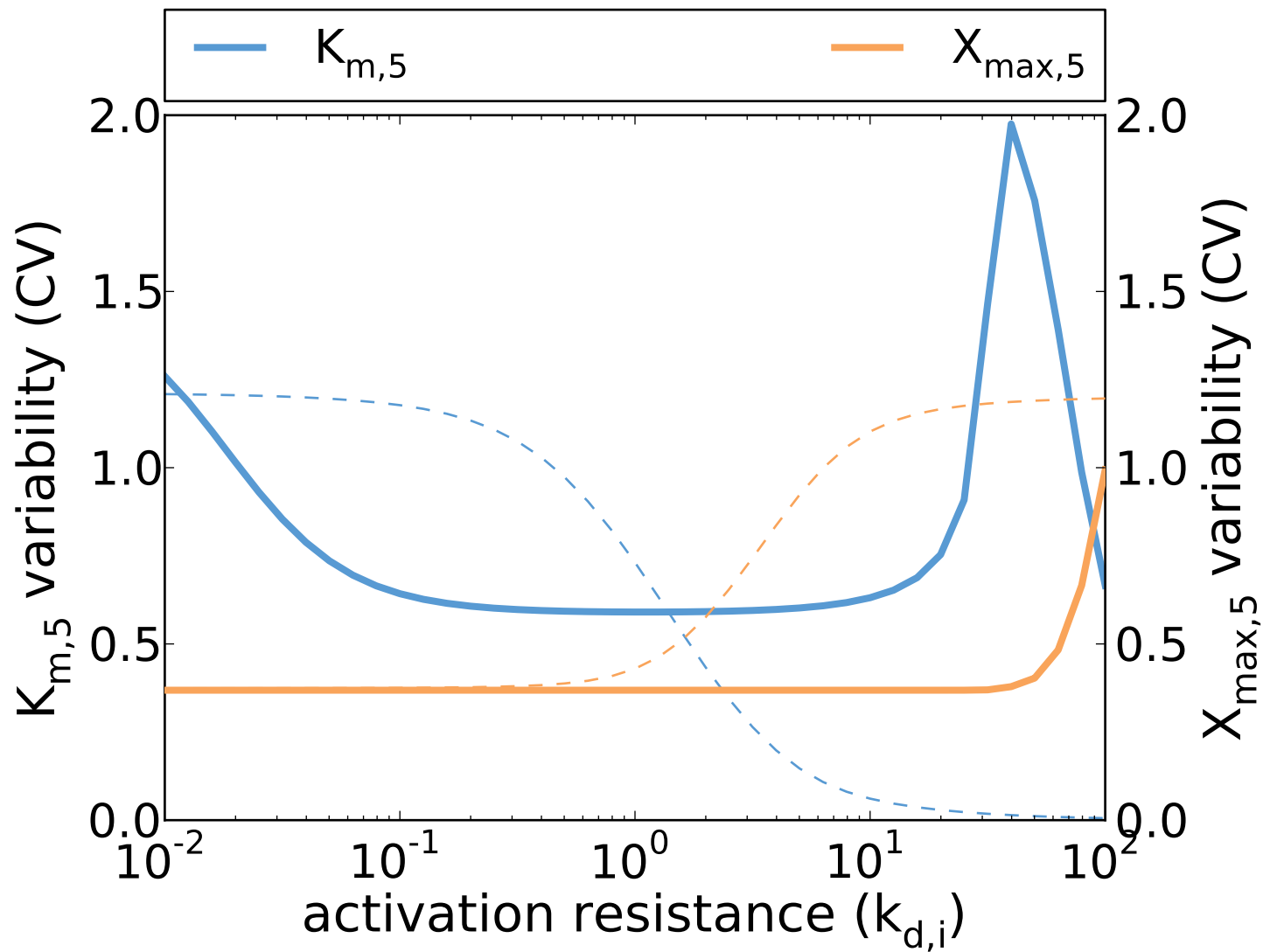

Supplement: Figure S6 — Cell-to-cell variability of ultrasensitive kinase cascades with feedforward regulation quantified using the coefficient of variation. The concepts are similar to Figure 6C, but the variabilities of and were analyzed using the coefficient of variation (CV = standard deviation/mean) and compared to the gradual model (thin, dashed lines). (PDF) [file pcbi.1003357.s006.pdf]

Real parts

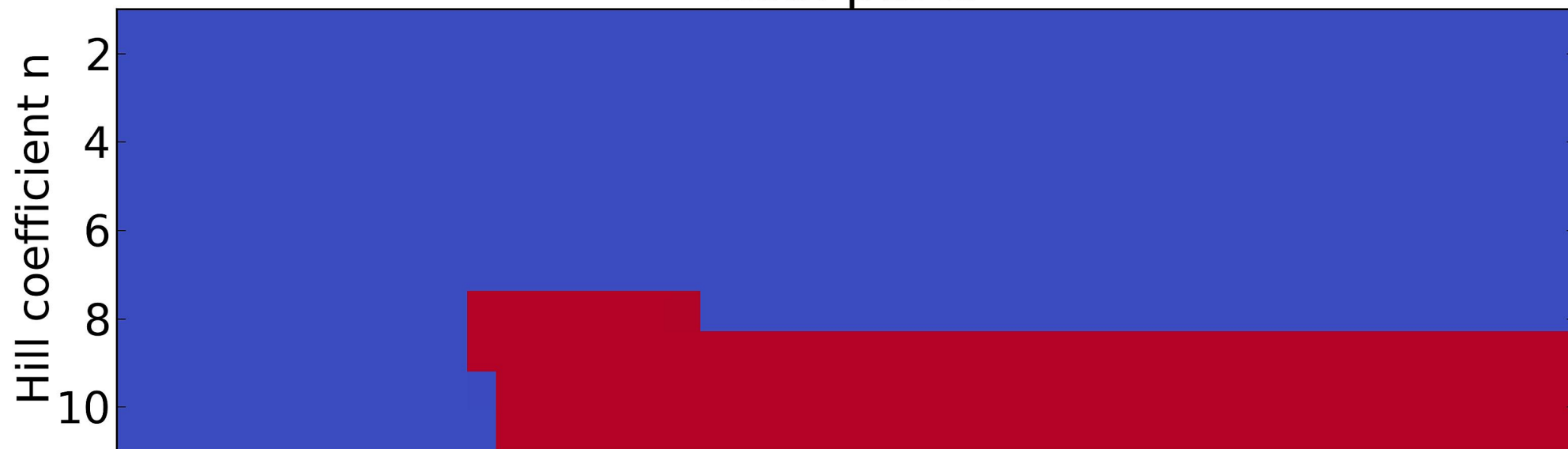

Imaginary parts

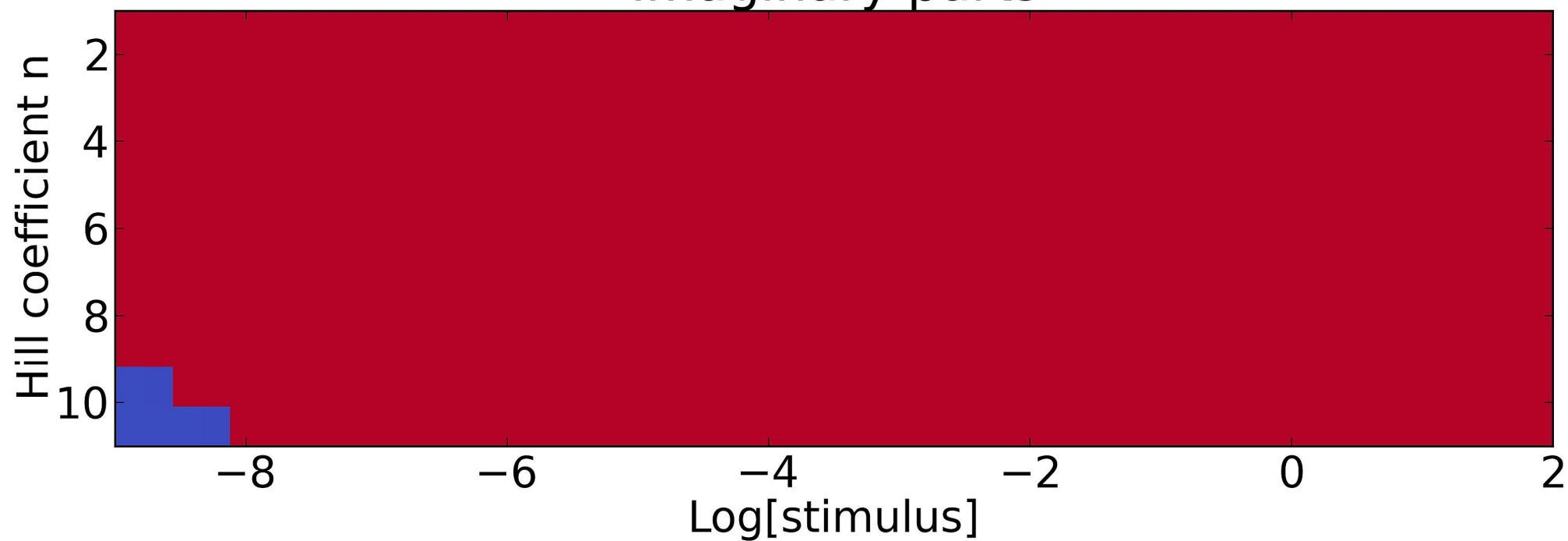

Supplement: Figure S7 — Numerical stability analysis of the upstream feedback cascade with low activation resistances reveals that high feedback cooperativity is required for sustained oscillations. The eigenvalues of the Jacobi matrix at the steady state were calculated numerically for different feedback cooperativities and stimuli as described in Supplemental Text S1. The upper panel shows a classification of the real parts of the eigenvalues (blue: real parts of all eigenvalues are negative; red: real part of at least one eigenvalue is zero or positive). The lower panel indicates whether at least one of the eigenvalues is complex (red regions) or not (blue regions). Oscillations require that at least one of the eigenvalues is complex (red regions, lower panel). A damped oscillator exhibits only negative real parts (blue regions, upper panel), while at least one real part is zero or positive for sustained oscillators (red regions, upper panel). Sustained oscillations require very strong feedback cooperativity, (). All activation resistances in the cascade were assumed to be low , Eq. 5, main text). The simulations cover the full dynamic range of the dose-response curves. Parameters: , , and , . (PDF) [file pcbi.1003357.s007.pdf]

Real parts

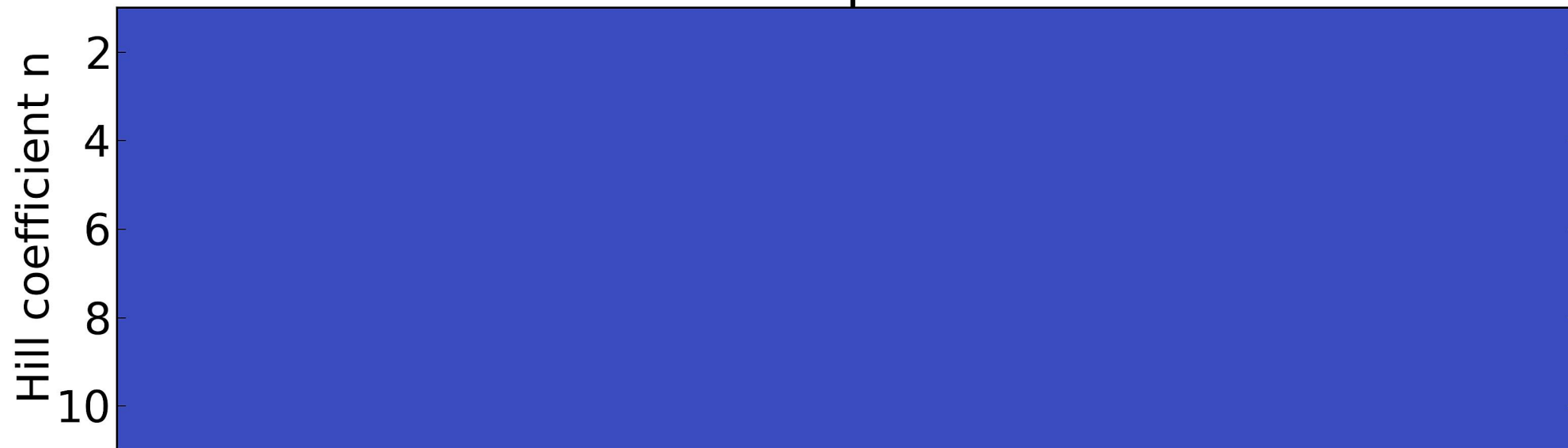

Imaginary parts

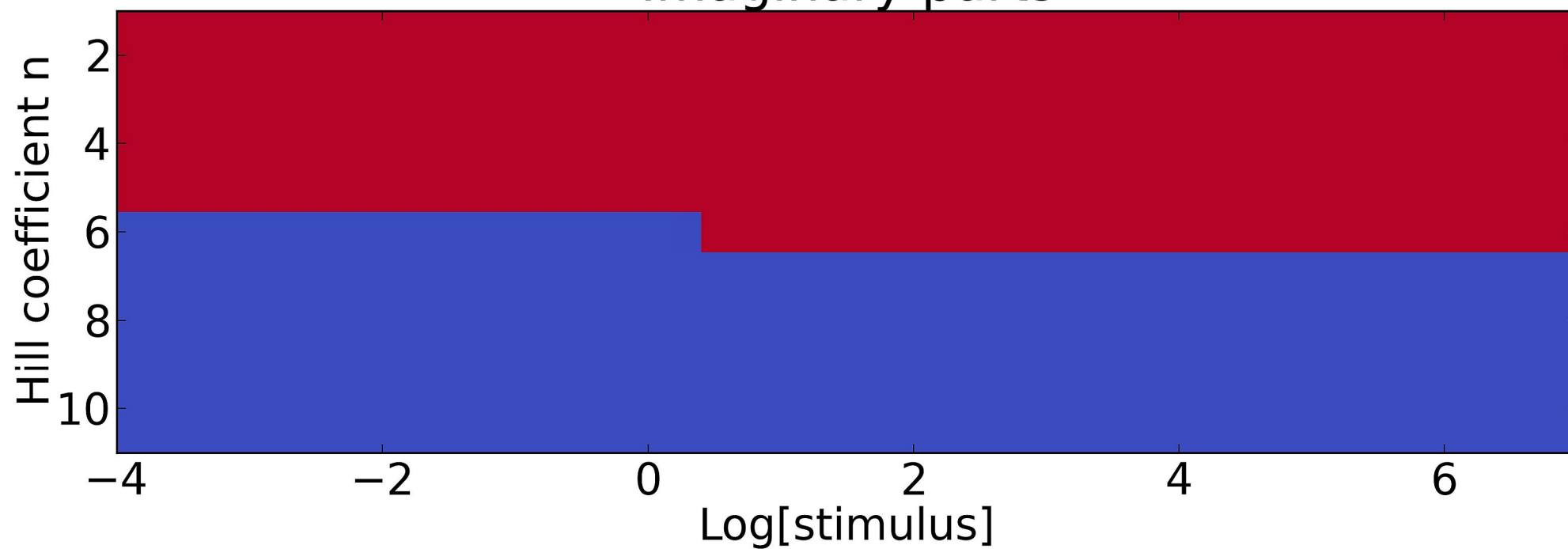

Supplement: Figure S8 — Numerical stability analysis of the upstream feedback cascade with high activation resistances reveals that sustained oscillations are not possible even for very strong feedback cooperativity. The concepts are similar to Figure S7, but all activation resistances in the cascade were assumed to be high (, Eq. 5, main text). Parameters: , , and , . (PDF) [file pcbi.1003357.s008.pdf]
