# Supplementary material for: Determinants of Cell-to-Cell Variability in Protein Kinase Signaling
Source: PLoS Comput Biol. 2013 Dec 5;9(12):e1003357. doi: 10.1371/journal.pcbi.1003357 (PMC3854479; doi:10.1371/journal.pcbi.1003357)
Supplement: Protocol S1 — Implementation of model variants. (PDF) [file pcbi.1003357.s009.pdf]

# Supplemental Protocol S1: Implementation of model variants

In the following, we briefly describe how each model variant was implemented. The parameter values used to generate the main text figures can be found in Supplemental Table S1.

The steady state behavior of the basic cascade model with linear kinetics (Eq. 1; Fig. 1A) was solved analytically, and the resulting equations (Eqs. 3 and 4) were used to generate Figs. 1B –D. The basic cascade model was extended to investigate the impact of negative feedback loops at different levels (Fig. 2A; Eqs. 8 and 12). The steady state behavior for very strong feedback (Fig. 2B, thin solid lines in Figs. 2C and D) was calculated using analytical approximations (Eqs. 9 and 13). The behavior of cascades with moderate feedback strength (thick lines in Figs. 2C and 2D) was characterized using numerical simulations of the ODE systems (Eqs. 8 and 12). Ultrasensitivity with distributed switching was introduced into the basic cascade model by assuming that the steady state dose-response curve at each level follows a Hill equation (similar to Eq. 14). The global dose-response behavior of the five-step signaling cascade with distributed ultrasensitivity was calculated by iteratively applying these Hill equations in tandem (Figs. 3A–E). In Fig. 3C, it was assumed that the fluctuations in the first kinase concentration ($X_{tot,1}$) and the antagonizing second phosphatase concentration ($P_{tot,2}$) are perfectly correlated (i.e., $X_{tot,1}$ and $P_{tot,2}$ were set equal). The global dose-response behavior of the ultrasensitive cascade with switching at a single step was calculated (Fig. 4) by iteratively applying a gradual Michaelis-Menten equation describing the fourth cascade level (Eq. 16) and a switch-like Hill equation describing the terminal kinase (Eq. 17). In Fig. 5, the ultrasensitive signaling cascade with switching at a single step (Eq. 16 and 17) was embedded in a transcriptional negative feedback loop by additionally considering an ordinary differential equation describing the expression of the phosphatase at the second level (Eqs. 18 or 19). The feedback system was solved by numerical integration: The basal state was simulated using Eq. 18 and assuming a very low stimulus level, while supra-basal acute stimulation was simulated using Eq. 19. Feedforward regulation was implemented in the ultrasensitive model with switching at a single step by replacing the original switch-like Hill equation describing the terminal level (Eq. 17) by a Hill equation taking into account that $X_1$ directly enhances the phosphorylation reaction of $X_5$ (Eq. 20). The simulations of the feedfoward system in Fig. 6 were performed by iteratively applying Michaelis-Menten and Hill equations in tandem (Eqs. 16 and 20).
