# Supplementary material for: Determinants of Cell-to-Cell Variability in Protein Kinase Signaling
Source: PLoS Comput Biol. 2013 Dec 5;9(12):e1003357. doi: 10.1371/journal.pcbi.1003357 (PMC3854479; doi:10.1371/journal.pcbi.1003357)
Supplement: Table S1 — Parameter values used for simulations. The index runs over the set if not specified explicitely. (PDF) [file pcbi.1003357.s010.pdf]

Supplemental Table S1: Parameters values used for simulations

Figures 1 and S1 (simulated using Eqs. 3 and 4 in the main text)

Parameter	Fig. 1B/1C	Fig. 1D/S1
$k_{a,i}$	1	1
$k_{d,1}$	1	1
$k_{d,i},\ i = 2, 3, 4$	0.1	$[10^{-2}, 10^{2}]$
$k_{d,5}$	0.1	0.1
$X_{tot,i}, P_{tot,i}$	$\text{Log}\mathcal{N}(0, 0.35)$	$\text{Log}\mathcal{N}(0, 0.35)$

Figures 2 and S2 (simulated using Eqs. 8, 9, 12 and 13 in the main text)

Parameter	Fig. 2B	Fig. 2C/2D/S2
$k_{a,i}$	1	1
$k_{d,1}$	1	1
$k_{d,i},\ i = 2, 3, 4$	0.1	$[10^{-2}, 10^{2}]$
$k_{d,5}$	0.1	0.1
$X_{tot,i}, P_{tot,i}$	$\text{Log}\mathcal{N}(0, 0.35)$	$\text{Log}\mathcal{N}(0, 0.35)$
k_{fb}	10^{15}	$10^{3}, 10^{15}$
n	1	1

Figures 3 and S3 (simulated using a five-step cascade similar to Eq. 14 in the main text)

Parameter	Fig. 3A/3B	Fig. 3C	Fig. 3D/S3	Fig. 3E
$k_{a,i}$	1	1	1	1
$k_{d,1}$	1	1	1	1
$k_{d,i},\ i = 2, 3, 4$	0.1	0.1	$k_{d,2} = [10^{-2}, 10^{2}]$<br>$k_{d,3} = k_{d,4} = 0.1$	$k_{d,2} = [0.45, 5.01, 112.2]$<br>$k_{d,3} = k_{d,4} = 0.1$
$k_{d,5}$	0.1	0.1	0.1	0.1
$X_{tot,i}, P_{tot,i}$	$\text{Log}\mathcal{N}(0, 0.35)$	$\text{Log}\mathcal{N}(0, 0.35)$<br>$X_{tot,1} = P_{tot,2}$	$\text{Log}\mathcal{N}(0, 0.35)$	$\text{Log}\mathcal{N}(0, 0.35)$
n	2	2	2	2

Figures 4 and S4 (simulated using Eqs. 16 and 17 in the main text)

Parameter	Fig. 4A	Fig. 4B/S4
$k_{a,i}$	1	1
$k_{d,1}$	1	1
$k_{d,i},\ i = 2, 3, 4$	0.1	$[10^{-2}, 10^{2}]$
$k_{d,5}$	0.1	0.1
$X_{tot,i}, P_{tot,i}$	$\mathrm{Log}\mathcal{N}(0, 0.35)$	$\mathrm{Log}\mathcal{N}(0, 0.35)$
n	5	5

Figures 5 and S5 (simulated using Eqs. 16–19 in the main text)

Parameter	Fig. 5B	Fig. 5C/S5
$k_{a,i}$	1	1
$k_{d,1}$	1	1
$k_{d,i},\ i = 2, 3, 4$	0.1	$[10^{-2}, 10^{2}]$
$k_{d,5}$	0.1	0.1
$X_{tot,i}, P_{tot,i}$	$\mathrm{Log}\mathcal{N}(0, 0.35)$	$\mathrm{Log}\mathcal{N}(0, 0.35)$
n	5	5
$v_{syn,const}$	10^{-2}	10^{-2}
$k_{syn,ind}$	10^{3}	10^{3}
k_{deg}	1	1

Figures 6 and S6 (simulated using Eq. 16 and 20 in the main text)

Parameter	Fig. 6B	Fig. 6C/S6
$k_{a,i}$	1	1
$k_{d,1}$	1	1
$k_{d,i},\ i = 2, 3, 4$	0.1	$[10^{-2}, 10^{2}]$
$k_{d,5}$	0.1	0.1
$X_{tot,i}, P_{tot,i}$	$\mathrm{Log}\mathcal{N}(0, 0.35)$	$\mathrm{Log}\mathcal{N}(0, 0.35)$
n	5	5
k_{FFL}	10^{5}	10^{5}
