# Supplementary material for: Determinants of Cell-to-Cell Variability in Protein Kinase Signaling
Source: PLoS Comput Biol. 2013 Dec 5;9(12):e1003357. doi: 10.1371/journal.pcbi.1003357 (PMC3854479; doi:10.1371/journal.pcbi.1003357)
Supplement: Text S1 — Analytical derivations and numerical analysis of steady state stability. (PDF) [file pcbi.1003357.s011.pdf]

Supplemental Text S1: Analytical derivations and numerical analysis of steady state stability

1 Modeling cell-to-cell variability

Cell-to-cell variability simulations were performed by drawing the kinase concentrations ($X_{tot,i}$) and the phosphatase concentrations ($P_{tot,i}$) from log-normal distributions (denoted $LogN$) with a mean μ and a variance σ^2, i.e.,

$$X_{tot,i} \sim \mathrm{Log}\mathcal{N}(\mu_{X_{tot,i}}, \sigma^2_{X_{tot,i}}),\ P_{tot,i} \sim \mathrm{Log}\mathcal{N}(\mu_{P_{tot,i}}, \sigma^2_{P_{tot,i}}). \quad (1)$$

We generally assumed the mean concentrations to be $\mu_{X_{tot},i} = \mu_{X_{tot},i} = 1$ and set the coefficient of variation (CV) to a value that represents typical intracellular protein expression noise (CV $= \sigma/\mu = 0.35$). Dose-response simulations were performed for each set of sampled protein concentrations using analytical equations or numerically (as indicated in the figure captions).

For certain cases, the signaling variability could calculated analytically: In a weakly stimulated signaling pathway, the activation resistances K_i, and thus the total kinase and phosphatase concentrations enter the signaling output in a multiplicative way (Eqs. 5 and 6, main text).

$$X_5 = X_{tot,5} \frac{S}{K_5 K_4 K_3 K_2 K_1} \quad (2)$$

The magnitude of cell-to-cell variability in the pathway output X_5 can be calculated by applying the standard laws for transforming random variables. The ratio of two log-normally distributed species is again a log-normal distribution with a variance that equals the sum of the individual variances, i.e.,

$$K_i = \frac{k_{d,i} P_{tot,i}}{k_{a,i} X_{tot,i-1}} \sim Log\mathcal{N}(\mu_{P_{tot,i}} - \mu_{X_{tot,i-1}} + \log(\frac{k_{d,i}}{k_{a,i}}), \sigma^2_{P_{tot,i}} + \sigma^2_{X_{tot,i-1}}) \quad (3)$$

Considering the denominator of Eq. 2 one derives the following expression for the distribution of X_5

$$\begin{aligned} \frac{1}{K_1 K_2 K_3 K_4 K_5} &\sim Log\mathcal{N}(-\log(\frac{k_{d,1}}{k_{a,1}}) - \sum_{i=2}^{5}(\mu_{P_{toti}} - \mu_{X_{tot,i-1}} + \log(\frac{k_{d,i}}{k_{a,i}})), \sum_{i=2}^{5}(\sigma^2_{P_{tot,i}} + \sigma^2_{X_{tot,i-1}})) \quad (4) \\ X_5 &\sim Log\mathcal{N}(\mu_{X_{tot,5}} - \log(\frac{k_{d,1}}{k_{a,1}}) - \sum_{i=2}^{5}(\mu_{P_{tot,i}} - \mu_{X_{tot,i-1}} + \log(\frac{k_{d,i}}{k_{a,i}})) + \log(S), \\ &\quad \sigma^2_{X_{tot,5}} + \sum_{i=2}^{5}(\sigma^2_{P_{tot,i}} + \sigma^2_{X_{tot,i-1}})) \end{aligned}$$

The total variance in the signaling output ($\sigma^2_{X_5}$) therefore equals the sum over all signaling protein concentration variances ($\sigma^2_{X_{tot,i}}$ for kinases and $\sigma^2_{P_{tot,i}}$ for phosphatases) as mentioned in Eq. 7 (main text).

$$\sigma^2_{X_5} = \sigma^2_{X_{tot,5}} + \sum_{i=2}^{5}(\sigma^2_{P_{tot,i}} + \sigma^2_{X_{tot,i-1}}). \tag{5}$$

Hence fluctuations in signaling protein levels are strongly amplified in a weakly stimulated cascade. The variability would is more pronounced for longer pathways. A similar reasoning was applied to derive an analytical expression for the cell-to-cell variability of the negative feedback system (Eq. 11, main text).

2 Sensitivity analysis reveals a trade-off in controlling pathway sensitivity and maximal activation

We applied one-dimensional sensitivity analysis to understand how fluctuations in certain kinase and phosphatase expression levels (i.e., in the parameters K_i and $X_{5,tot}$) affect the features of the dose-response curve in the gradual signaling cascade (Eq. 1, main text). The sensitivity of a signaling feature X with respect to a parameter or concentration change Y is typically quantified using the so-called control coefficient (a.k.a. gain): $C^X_Y = \frac{Y}{X} \cdot \frac{dX}{dY}$. The control coefficient is a normalized slope that quantifies how a relative change in Y translates into a relative change in X. A control coefficient of unity refers to a linear relationship, while $C^X_Y < 1$ and $C^X_Y > 1$ imply sub-linear and ultrasensitive behavior, respectively.

The sensitivities of the half-maximal pathway stimulus $K_{m,5}$ can be calculated using Eq. 4 (main text), and are given by

$$\begin{aligned} C^{K_{m,5}}_{K_1} &= 1 \\ C^{K_{m,5}}_{K_2} &= \frac{1 + K_5 + K_5K_4 + K_5K_4K_3}{1 + K_5 + K_5K_4 + K_5K_4K_3 + K_5K_4K_3K_2} \end{aligned} \tag{6}$$

$$\begin{aligned} C^{K_{m,5}}_{K_3} &= \frac{1 + K_5 + K_5K_4}{1 + K_5 + K_5K_4 + K_5K_4K_3 + K_5K_4K_3K_2} \\ C^{K_{m,5}}_{K_4} &= \frac{1 + K_5}{1 + K_5 + K_5K_4 + K_5K_4K_3 + K_5K_4K_3K_2} \end{aligned} \tag{7}$$

$$\begin{aligned} C^{K_{m,5}}_{K_5} &= \frac{1}{1 + K_5 + K_5K_4 + K_5K_4K_3 + K_5K_4K_3K_2} \\ C^{K_{m,5}}_{X_{5,tot}} &= 0 \end{aligned} \tag{8}$$

Similarly, one derives for the maximal pathway activation sensitivities

$$\begin{aligned} C^{X_{max,5}}_{K_1} &= 0 \\ C^{X_{max,5}}_{K_2} &= \frac{K_5K_4K_3K_2}{1 + K_5 + K_5K_4 + K_5K_4K_3 + K_5K_4K_3K_2} \end{aligned} \tag{9}$$

$$\begin{aligned} C^{X_{max,5}}_{K_3} &= \frac{K_5K_4K_3 \cdot (1 + K_2)}{1 + K_5 + K_5K_4 + K_5K_4K_3 + K_5K_4K_3K_2} \\ C^{X_{max,5}}_{K_4} &= \frac{K_5K_4 \cdot (1 + K_2 + K_3K_2)}{1 + K_5 + K_5K_4 + K_5K_4K_3 + K_5K_4K_3K_2} \end{aligned} \tag{10}$$

$$C_{K_5}^{X_{max,5}} = \frac{K_5 \cdot (1 + K_4 + K_4K_3 + K_4K_3K_2)}{1 + K_5 + K_5K_4 + K_5K_4K_3 + K_5K_4K_3K_2} \tag{11}$$
$$C_{X_{5,tot}}^{X_{max,5}} = 1$$

The sensitivity analysis reveals that fluctuations in signaling protein levels generally affect the dose-response features in a linear or less-than linear manner (all $C^{K_{m,5}} \leq 1$ and all $C^{X_{max,5}} \leq 1$). In terms of cell-to-cell variability, this means that signaling protein fluctuations tend to be dampened. $K_{m,5}$ is primarily controlled by the kinetic parameters and signaling protein concentrations regulating the upstream cascade steps ($C_{K_1}^{K_{m,5}} \geq C_{K_2}^{K_{m,5}} \geq C_{K_3}^{K_{m,5}} \geq C_{K_4}^{K_{m,5}} \geq C_{K_5}^{K_{m,5}}$). In contrast, $X_{max,5}$ is controlled more strongly by the features of the downstream cascade levels ($C_{K_1}^{X_{max,5}} \leq C_{K_2}^{X_{max,5}} \leq C_{K_3}^{X_{max,5}} \leq C_{K_4}^{X_{max,5}} \leq C_{K_5}^{X_{max,5}}$). Thus, $K_{m,5}$ and $X_{max,5}$ are controlled in an opposite way, and both cannot be made invariant at the same time by reducing the expression noise of a certain signaling protein (equivalent to reducing the fluctuations of a certain K_i).

The sensitivies and thus cell-to-cell variability may also be tuned by changing the kinetic rate constants of (de)phosphorylation ($k_{a,i}$ and $k_{d,i}$) or the median kinase/phosphatase concentrations, all of which determine the median values of the K_i. Increasing the K_i individually or simultaneously decreases the variability of $K_{m,5}$ (all $C_{K_i}^{K_{m,5}}$ decrease), but increases the variability of $X_{max,5}$ (all $C_{K_i}^{X_{max,5}}$ increase). $K_{m,5}$ and $X_{max,5}$ are therefore again controlled in an opposite way, and both cannot be made invariant at the same time by parameter tuning.

3 Analytical approximation for strong negative feedback regulation in a gradual kinase cascade

Strong feedback regulation ($k_{fb} \gg 1$) was assumed to allow for the derivation of analytical approximations for the cascade models with upstream or downstream negative feedback (Eqs. 9 and 13, main text). For both feedback variants, the steady state equation for X_5 can be written as a polynomial of degree $n+1$ (using Eqs. 8 and 12, main text):

$$0 = -ak_{fb}X_5^{n+1} - bX_5 + c, \tag{12}$$

with

$$\begin{aligned} a &= k_{d,5}K_2K_3K_4(S + K_1) \\ b &= k_{d,5}K_1K_2K_3K_4 + S(k_{d,5} + k_{a,5}\hat{X}_4 + K_4(k_{d,5} + K_3(k_{d,5} + K_2k_{d,5}))) \\ c &= k_{a,5}\hat{X}_4\hat{X}_5 \end{aligned} \tag{13}$$

for the second level feedback variant and

$$\begin{aligned} a &= k_{d,5}(K_1K_2K_3K_4 + S(1 + K_4(1 + K_3(1 + K_2)))) \\ b &= k_{a,5}\hat{X}_4S \\ c &= k_{a,5}\hat{X}_4\hat{X}_5 \end{aligned} \tag{14}$$

for the fifth level feedback variant. For strong feedback ($k_{fb} \gg 1$), the second term is small compared to the first one, and the steady state of X_5 can be approximated by:

$$X_5 \approx \sqrt[n+1]{\frac{c}{ak_{fb}}}. \tag{15}$$

Inserting Eqs. 13 and 14 into Eq. 15 allows to find the analytical approximations of feedback variants (Eqs. 9 and 13, main text).

4 Dose-response curve of an ultrasensitive two-step cascade with distributed switching

The two-step signaling cascade with distributed switching is described by Eq. 14 in the main text. Assuming the same Hill coefficient for both levels and a low phosphatase activity at the second level ($\tilde{K}_2 \ll X_{tot,1}$), one derives the following expression for the overall response of the two-stage cascade

$$X_2 = X_{tot,2} \frac{S^{n^2}}{S^{n^2} + \tilde{K}_1^{n^2} \frac{\tilde{K}_2^n}{X_{tot,1}^n}}. \tag{16}$$

The overall dose-response curve has the form of the Hill equation, and the high Hill coefficient (n^2) indicates amplification of ultrasensitivity. The maximal activation level equals the total concentration of the terminal kinase ($X_{max,2} = X_{tot,2}$) and the half-saturation point where the system switches from low to high activation is given by Eq. 15 in the main text.

5 Threshold variability in an ultrasensitive signaling cascade with switching at a single step

The ultrasensitive signaling cascade with switching at a single step is described by Eqs. 16 and 17 in the main text. The parameters controlling the heterogeneity of the threshold stimulus ($K_{m,5}$) can be estimated by setting $X_4 = \tilde{K}_5$ in Eq. 16 (main text). Solving for the stimulus S we obtain:

$$K_{m,5} = \frac{K_{m,4}\tilde{K}_5}{X_{max,4} - \tilde{K}_5} \approx \frac{K_{m,4}\tilde{K}_5}{X_{max,4}}. \tag{17}$$

In this approximation, we assumed that the maximal activation of X_4 significantly exceeds the Hill equation threshold ($X_{max,4} \gg \tilde{K}_5$) . The overall threshold of the signaling cascade is thus proportional to the local threshold of the Hill equation ($\tilde{K}_5$) times the ratio $X_{max,4}/K_{m,4}$ which depends on the product $K_4K_3K_2K_1$ in a gradual kinase cascade (cf. Eq. 6, main text). In total, seven protein concentrations enter the signaling threshold in a multiplicative fashion: the phosphatase determining the Hill equation threshold, and three kinase-phosphatase pairs controlling the gradual upstream cascade (K_2–K_4). As a conclusion, a signaling cascade with a localized switch at the terminal level will always show pronounced variability in the signaling threshold.

6 Basal transcriptional feedback corrects for pathway threshold fluctuations

Slow transcriptional feedback may measure pre-stimulation basal signaling to suppress the signaling threshold variability, without necessarily affecting the steepness of the dose-response curve upon acute stimulation (Fig. 5B, main text). In the following, it will be shown that basal state signaling activity and the signaling threshold depend on the same combination of kinetic parameters and protein concentrations. A mechanism that suppresses the variability of basal state signaling is therefore (potentially) able to reduce signaling threshold fluctuations as well.

The basal state activity of the ultrasensitive pathway (Eqs. 16 and 17, main text) is given by

$$X_5 \approx X_{tot,5} \frac{X_{max,4}^n S_b}{\tilde{K}_5^n K_{m,4}^n} = \frac{X_{tot,5} S_b}{K_{m,5}^n}. \tag{18}$$

Here it is assumed that a very low basal stimulus $S_b \ll K_{m,4}$ is present and that the basal activation of X_4 is much lower than the Hill equation threshold ($\tilde{K}_5$). The basal state signaling activity in Eq. 18 strongly

depends on the threshold stimulus concentration $K_{m,5}$ where the terminal kinase level is switched on (Eq. 17) and to a much lesser extent on the total concentration of the fifth kinase ($X_{tot,5}$). A transcriptional negative feedback driven by basal signaling could therefore efficiently correct for imbalances in the pathway activation threshold, e.g. by enhancing the expression of a phosphatase acting upstream of the switch. The expression level of this induced phosphatase would be higher for low signaling thresholds (i.e., for higher basal signaling). Variability suppression would be very efficient because the signaling threshold enters Eq. 18 with a high exponent n, giving rise to strong nonlinearity in the basal transcriptional feedback loop.

7 Analysis of oscillatory behavior for kinase cascades with negative feedback regulation

Negative feedback regulation is known to establish oscillations in biochemical networks if the feedback is sufficiently strong and nonlinear, and additionally characterized by a delay. In the following, we employ linear stability analysis and numerical simulations to analyze whether the negative feedback circuits in this paper can show damped or sustained oscillations. In the case of damped oscillations, the systems reach a steady state after a transition time with oscillatory behavior, while they continue to oscillate around the steady state if oscillations are sustained. The cell-to-cell variability simulations in this paper reflect the steady state behavior for non-oscillatory systems and for damped oscillators. For sustained oscillations, the simulations show the cell-to-cell variability in the mean activation level of the oscillator.

Stability analysis of the steady state $\mathbf{X_{ss}}$ of a dynamic system $\dot{\mathbf{X}} = f(\mathbf{X})$ is done by linearization using the Jacobi matrix D_f.

$$f(\mathbf{X}) = D_f(\mathbf{X_{ss}}) \cdot (\mathbf{X} - \mathbf{X_{ss}}) + f(\mathbf{X_{ss}}) \tag{19}$$

$D_f(\mathbf{X_{ss}})$ is the Jacobi matrix at the steady state and the entries of this matrix are defined as $D_{f,ij} = \frac{\partial \dot{X}_i}{\partial X_j}$. The expression $D_f(\mathbf{X_{ss}}) \cdot (\mathbf{X} - \mathbf{X_{ss}})$ equals the linear term of a Taylor expansion around the steady state. The eigenvalues of D_f allow statements about the dynamics of the system and about the stability of the steady state. Oscillatory behavior can only be observed if at least one of the eigenvalues is complex. The stability is determined by the real parts of the eigenvalues: The steady state $\mathbf{X_{ss}}$ is stable if all real parts are negative and unstable if at least one of the real parts is positive. A damped oscillator exhibits only negative real parts, while at least one real part is zero or positive for sustained oscillators.

The eigenvalues λ_i of the gradual system without negative feedback (Eq. 1, main text) can be calculated analytically:

$$\lambda_i = -k_{d,i} P_{tot,i} - k_{a,i} X_{i-1_{SS}} \leq 0 \tag{20}$$

These eigenvalues are real valued, implying that no oscillations around the steady state are possible. The steady state is stable, because all eigenvalues are all smaller than zero, thus confirming the hypothesis oscillations cannot be observed without negative feedback regulation.

Next, we investigated the gradual cascade with downstream negative feedback, where X_5 activates its own phosphatase P_5 (Eq. 12, main text). The eigenvalues of the Jacobian are

$$\begin{aligned} \lambda_i &= -k_{d,i} P_{tot,i} - k_{a,i} X_{i-1_{SS}}, \quad i = 1 \ldots 4 \\ \lambda_5 &= -k_{d,5} P_{tot,5} - k_{a,5} X_{i-5_{SS}} - k_{d,5} k_{fb} (n+1) P_{tot,5} X_{5_{SS}}^n \end{aligned} \tag{21}$$

Again, all eigenvalues are real and smaller than zero for biologically relevant parameter ranges. Thus, the system with a short, downstream negative feedback does not exhibit damped or sustained oscillations even for strongly cooperative feedback ($n \gg 1$). This result is consistent with previous findings [1]. Oscillations are absent, because the feedback loop does not exhibit a significant delay.

In contrast, a delay and thus oscillations can be observed in the system with upstream feedback (Eq. 8, main text), as the feedback mediator (X_5) is separated from the feedback site of action (P_2) by several steps. The eigenvalues of the system with upstream feedback could not be solved analytically. We therefore analyzed the steady state stability using numerical simulations: The steady state of the system was calculated

by numerically integrating the ODE system (Eq. 8, main text). The simulation result was used to formulate the Jacobian D_f at the steady state, and the corresponding eigenvalues were calculated numerically.

We investigated how strong the feedback cooperativity needs to be to observe oscillations in some parts of the dose-response curve. The eigenvalues are thus shown for variations in the feedback cooperativity n and the stimulus S (Figs. S7 and S8). For simplicity, we assumed strong feedback ($k_{fb} = 10^{15}$), and fixed the kinase rates $k_{a,i}$ as well as the total kinase and phosphatase concentration in the cascade ($X_{tot,i}$ and $P_{tot,i}$, respectively) to 1. Stability analyses were performed for two phosphatase activity levels in the cascade: In Fig. S7, it was assumed that all activation resistances in the cascade are low ($K_i = 0.1$, Eq. 5, main text) by setting $k_{d,i} = 0.1$. In Fig. S8, all activation resistances in the cascade were high ($K_i = 10$, Eq. 5, main text) by setting $k_{d,i} = 10$.

The cascade with low activation resistances (Fig. S7) shows complex eigenvalues over a broad range of stimuli and feedback cooperativities (red area, lower panel). Sustained oscillations require very strong feedback cooperativity, because the real parts of all eigenvalues are negative unless $n > 7$ (red area, upper panel). We conclude the corresponding cell-to-cell variability simulations reflect the steady state behavior of a non-oscillatory system or a damped oscillator as long as $n < 7$.

The cascade with high activation resistances (Fig. S8) shows complex eigenvalues over a broad range of stimuli and feedback cooperativities as well (red area, lower panel), but the real parts are always negative (red area, upper panel). We conclude that the system does not show sustained oscillations in the analyzed parameter regime, most likely because the high phosphatase activity at the second cascade level prevents that negative feedback affects the system dynamics significantly. The corresponding cell-to-cell variability simulations thus reflect the steady state behavior of a non-oscillatory system or a damped oscillator.

The gradual cascade with upstream negative feedback resembles the Goodwin oscillator. This classical model biochemical comprises mRNA synthesis, mRNA translation into protein and feedback inhibition of transcription by the biologically active protein product [2]. Previous studies have shown that a Goodwin oscillator with four steps between mRNA transcription and formation of the mature protein can only show sustained oscillation for a cooperativity factor of $n > 4$ [2, 1]. We find that higher cooperativities are required in our analogous system which involves four steps in a kinase cascade, most likely because mass conservation effects in phosphorylation-dephosphorylation cycles lead to saturation effects, thereby reducing the efficiency of negative feedback regulation [1].

References

[1] Kholodenko BN (2000) Negative feedback and ultrasensitivity can bring about oscillations in the mitogen-activated protein kinase cascades. Eur J Biochem 267: 1583–1588.

[2] Fall CP, Marland ES, Wagner JM, Tyson JJ, editors (2002) Computational Cell Biology. Springer.
